# Olefin Metathesis in Continuous Flow Reactor Employing Polar Ruthenium Catalyst and Soluble Metal Scavenger for Instant Purification of Products of Pharmaceutical Interest

**DOI:** 10.1021/acssuschemeng.1c06522

**Published:** 2021-11-22

**Authors:** Ren Wei Toh, Michał Patrzałek, Tomasz Nienałtowski, Jakub Piątkowski, Anna Kajetanowicz, Jie Wu, Karol Grela

**Affiliations:** † Department of Chemistry, National University of Singapore, 3 Science Drive 3, Singapore 117543, Singapore; ‡ Biological and Chemical Research Centre, Faculty of Chemistry, University of Warsaw, Żwirki i Wigury 101, 02-089 Warsaw, Poland; § Pharmaceutical Works Polpharma SA, Pelplińska 19, 83-200 Starogard Gdański, Poland

**Keywords:** Flow chemistry, Olefin metathesis, Ring-closing metathesis, Scavenger, Ruthenium, Green chemistry

## Abstract

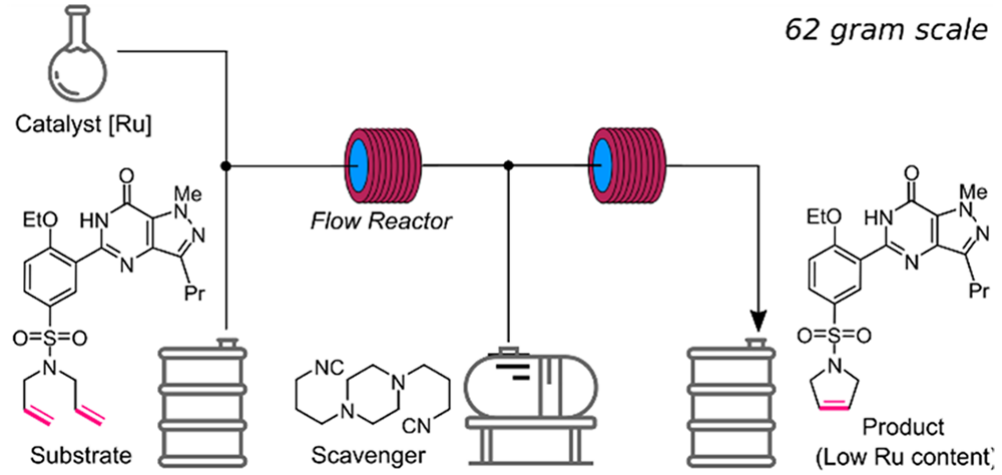

In recent years, the development of continuous-flow reactors has attracted growing attention from the synthetic community. Moreover, findings in the precise control of the reaction parameters and improved mass/heat transfer have made the flow setup an attractive alternative to batch reactors, both in academia and industry, enabling safe and easy scaling-up of synthetic processes. Even though a majority of the pharmaceutical industry currently rely on batch reactors or semibatch reactors, many are integrating flow technology because of easier maintenance and lower risks. Herein, we demonstrate an operationally simple flow setup for homogeneous ring-closing metathesis, which is applicable to the synthesis of active pharmaceutical ingredients precursors or analogues with high efficiency, low residence time, and in a green solvent. Furthermore, through the addition of a soluble metal scavenger in the subsequent step within the flow system, the level of ruthenium contamination in the final product can be greatly reduced (to less than 5 ppm). To ensure that this method is applicable for industrial usage, an upscale process including a 24 h continuous-flow reaction for more than 60 g of a Sildenafil analogue was achieved in a continuous-flow fashion by adjusting the tubing size and flow rate accordingly.

## Abstract 2

Olefin metathesis using StickyCat catalyst and SnatchCat metal scavenger provided valuable products with low Ru content on a larger scale.

## Introduction

**Figure 1:**
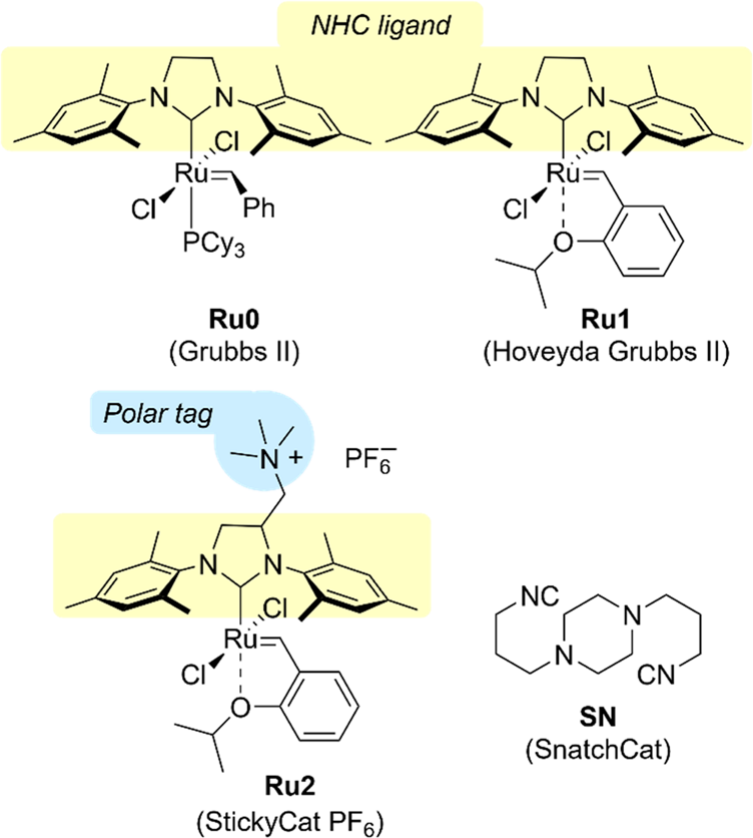
Grubbs (**Ru0**), Hoveyda–Grubbs (**Ru1**) and its polar onium-tagged analogue (**Ru2**) 2nd generation olefin metathesis catalysts, and metal scavenger SnatchCat (**SN**).

One of the objectives in organic synthesis is the production of complex polyfunctional bioactive drug molecules with high purity. In recent years, the usage of catalysts based on rare earth metals is common for the synthesis of active pharmaceutical ingredients (API) in the pharmaceutical industry.^1−4^ One such process that is finding more and more applications in the synthesis of biologically active compounds is olefin metathesis.^5−7^ It enables the formation of new C–C double bonds and relies mainly on the complexes of two transition metals, ruthenium and molybdenum.^8−10^ The development of modern catalysts, especially Grubbs and Hoveyda–Grubbs second-generation Ru-complexes (Figure 1), as well as their polar analogues that are easier to be separated after the reaction (like StickyCat PF_6_, Figure 1), has significantly facilitated the synthesis of even complex organic compounds and enabled a substantial reduction of the catalyst loading.^11^ Nevertheless, the reliance on heavy metals for organic synthesis potentially leads to metal contamination because traditional purification methods like column chromatography and recrystallization is inefficient in purifying complex polyfunctional chemical substances such as APIs in downstream processes.^12^ Additionally, in some cases, ruthenium residues in the product can cause isomerization (migration over one or more positions in a hydrocarbon chain) of the double bond, leading to complex reaction mixtures that are difficult to be separated.^13−15^ Therefore, in recent years, different approaches have been employed to help counter the problem of metal contamination, especially in large-scale chemical production of drugs and complex natural products.

The main approaches to reduce the heavy metal content are^12,16,17^ (1) reduction of catalyst loading; (2) conventional purification methods such as recrystallization, distillation, chromatography, and nanofiltration;^18−21^ (3) addition of scavengers into postreaction mixtures;^22−24^ (4) self-scavenging catalysts being complexes with modified ligands containing an additional functional group;^25,26^ (5) usage of heterogeneous catalysts.^27−29^ The latter was not utilized in our research because of typical problems which may occur, such as metal leaching, low catalytic efficiency, and clogging, particularly in a flow system. There is minimal information about heterogeneous catalysts being utilized in the industrial setting.^30^

Typically, the most commonly used metal scavengers in the context of olefin metathesis were water-soluble phosphines^31,32^ and phosphine oxides.^24^ In 2007, Diver’s group synthesized a scavenger which contains an isocyanide group that can bind to residual ruthenium allowing for easier product purification via column chromatography.^33^ Building on this foundational concept, we developed a scavenger incorporating two isocyanide groups that can bind efficiently to ruthenium metal, hence reducing the ruthenium content to less than 1 ppm with the usage of minimal amount of silica gel.^22,34−36^

### Flow Chemistry

Continuous-flow chemistry has gained much attention in the past decade because of its several advantages over batch systems.^37−40^ Continuous manufacturing has been applied in petrochemical and bulk chemical industries for a number of years. However, the synthesis of APIs is usually carried out in batch or semibatch reactors becaues of their similarities to reactions in R&D laboratories which are carried out in test tubes, round-bottom flasks, or closed vessels. However, in 2019, with the encouragement of the FDA to improve drug quality while reducing the environmental impact, GlaxoSmithKline opened their first continuous plant in Singapore.^41^ Other pharmaceutical companies such as Novartis, Johnson and Johnson, and Vertex Pharmaceuticals also built plants for continuous-flow reactions to embrace this technology.

The reason for the migration toward continuous-flow plants is because a smaller platform is required for large-scale production compared with batch reactors.^42,43^ For instance, in 2013, Trout and co-workers demonstrated the synthesis of Aliskiren in a 100 g/hour scale using the continuous-flow system. In comparison to the normal batch reactor which requires 1500 L of reactor volume, the estimated reactor volume to produce the same mass of product in this continuous-flow setup is only 136 L. Hence, the reduction in the carbon footprint and the waste solvent volume (usually burnt) makes continuous-flow synthesis highly attractive to researchers and industries.

Moreover, in a typical flow setup, the reactions are conducted in microtubing reactors.^37−40^ This allows highly exothermic reactions to be carried out in a safer manner as only a small volume of different reagents can mix in the reactor during the synthesis, mitigating safety issues such as overpressure or overheating, which may occur while using large batch reactors. Furthermore, the usage of microtubing reactors enhances heat and mass transfer, allowing for a shorter residence time compared with a batch reactor.^44−46^

By incorporating a back-pressure regulator (BPR) at the end of the tubing, the pressure in the reactor can be easily controlled, enabling superheating of solvents beyond their boiling points. Moreover, in a continuous-flow setup, the flow rate of the reagents can be tuned easily, allowing better and more precise control on the addition of building blocks to a substrate. For example, in 2019, Wu’s group had demonstrated that by tuning the time and temperature in a continuous-flow setup with dichloromethane as both the solvent and chlorine source, the number of chlorine atoms substituted to silane could be precisely controlled.^47^

Since 2012, olefin metathesis has been applied in continuous-flow reactors.^48−50^ Their primary focus was on different kinds of flow reactor technical designs, such as using super critical carbon dioxide^51^ or dimethyl carbonate as a solvent,^52^ tube-in-tube system for removal of ethylene gas,^53^ addition of argon or nitrogen for ethylene exchange,^54^ immobilization of homogeneous catalyst to reduce ruthenium content,^27,28^ and usage of membrane to trap catalyst.^55,56^ However, in those cases, the ability to upscale is limited due to the sophisticated and customized design. Furthermore, the usage of polar solvents with heterogeneous catalysts resulted in severe leaching issues, which limited the substrate scope in the proposed systems.^27,28^

Herein, we report a highly efficient ring-closing metathesis (RCM) in flow that uses the commercially available homogeneous StickyCat PF_6_ catalyst^57^ and SnatchCat scavenger (1,4-bis(3-isocyanopropyl)piperazine)^22,34−36^ to continuously produce advanced functionalized products, resembling in their complexity selected API substances, with low ruthenium content. With this system, without ethylene removal, we conducted a number of RCM reactions of highly functionalized substrates and managed to upscale the flow process to produce 62 g of a Sildenafil analogue in 24 h with a very low ruthenium content of 0.5 ppm with an environmental-friendly green solvent, ethyl acetate.

## Results and Discussion

### Selection of Catalyst and Solvent

To date, most of the published olefin metathesis reactions were performed in benzene, toluene, dichloromethane, or chloroform as solvents. Unfortunately, owing to the detrimental effect of these reaction media on the environment (dichloromethane and 1,2-dichloroethane belong to ICH class 1 and toluene to ICH class 2 solvents),^58^ their use, especially in industrial processes, is restricted or even prohibited. Green chemistry is an increasing trend not only in chemical industry but also in academia.^59−62^ Despite the environmental needs and the availability of many alternative solvents such as water,^63^ ethyl acetate,^64,65^ dimethyl carbonate,^66,67^ 2-MeTHF,^68,69^ and 4-MeTHP,^61,70^ the usage of green solvent for olefin metathesis is relatively scarce. We therefore screened both the general-purpose Hoveyda–Grubbs second-generation catalyst (**Ru1**, see Figure 1) and the tagged StickyCat PF_6_ (**Ru2**) in RCM of diethyl diallylmalonate (**1**) used as a model substrate, in two solvent systems—DCM (not green) and EtOAc (green).

**Figure 2:**
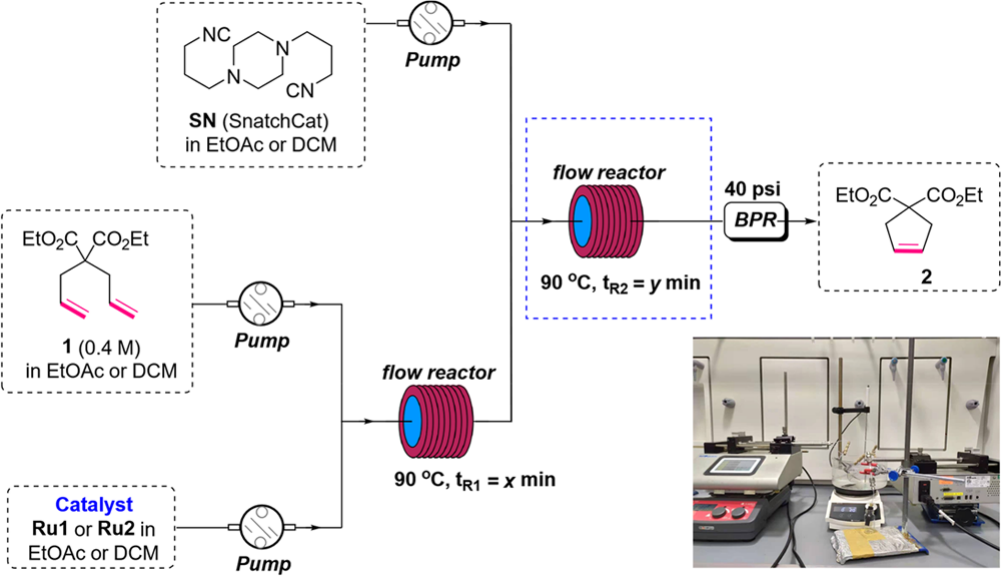
Representative schematic flow setup of ring-closing metathesis of diethyl diallylmalonate (**1**) with **Ru1** or **Ru2** for optimization.

Prior to testing the reactions in flow, the activities of two selected catalysts in two solvents were tested in batch. As shown in Table 1, a considerable difference in reactivity between Hoveyda–Grubbs (**Ru1**) and StickyCat PF_6_ (**Ru2**) complexes was observed, especially at lower catalyst loadings (entries 1–6). Interestingly, only minimal differences were observed between the two solvents (entries 1 and 4). Next, the reaction conditions were investigated in a flow reactor at higher temperature by taking advantage of the flow technique such as superheating of the reaction mixture (schematic setup shown in Figure 2). It was observed that at 90 °C, the reactivities of both catalysts were similar (entries 7 vs 13, 8 vs 15). Similar to batch conditions, the solvent effect on the reactivity was minimal in flow. The RCM of **1** performed in the presence of **Ru2** in EtOAc at 90 °C in a flow reactor required higher catalyst loading than that for the batch reaction (0.3 mol % versus 0.1 mol %); however, it allowed for an increase of yield as well as a significant reduction of reaction time from 3 h to 2.5 min.

**Table 1:**
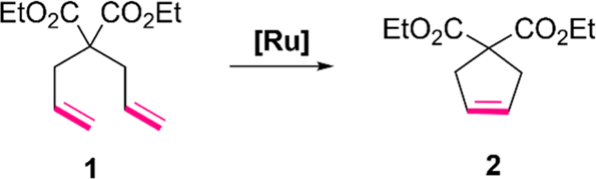
Comparison of RCM Reaction of **1** in the Presence of **Ru1** and **Ru2** Performed in DCM or EtOAc in Both Batch and Flow Reactors^a^

entry	reactor	solvent	catalyst	loading (mol %)	temperature (°C)	*t*_R1_ (min)	yield (%)^b^
1	batch	DCM	**Ru1**	0.1	30	180	83
2	batch	EtOAc	**Ru1**	1.0	30	30	94
3	batch	EtOAc	**Ru1**	0.1	30	180	31
4	batch	DCM	**Ru2**	0.1	30	180	81
5	batch	EtOAc	**Ru2**	1.0	30	30	96
6	batch	EtOAc	**Ru2**	0.1	30	180	33
7	flow	EtOAc	**Ru1**	0.3	90	2.5	98
8	flow	DCM	**Ru1**	0.3	90	2.5	93
9	flow	EtOAc	**Ru2**	0.1	30	1.5	1
10	flow	EtOAc	**Ru2**	0.1	90	1.5	49
11	flow	EtOAc	**Ru2**	0.2	90	1.5	73
12	flow	EtOAc	**Ru2**	0.3	90	1.5	73
13	flow	EtOAc	**Ru2**	0.3	90	2.5	94
14	flow	EtOAc	**Ru2**	0.5	90	2.5	93
15	flow	DCM	**Ru2**	0.3	90	2.5	93

a Conditions: For reactions performed in batch *C*_M(**1**)_ = 0.1 M; for reactions performed in flow *C*_M(**1**)_ = 0.4 M.

b Yield was determined by ^1^H NMR spectroscopy (for flow reactions) or GC measurement (for batch reactions).

From the results obtained in the flow system, it was observed that low catalyst loading (0.1 mol % of StickyCat PF_6_) combined with short residence time (*t*_R1_ = 1.5 min) led to moderate reactivity (entry 10). Slightly better activity, leading to a 73% yield of **2**, was observed when the loading was doubled. Further increase of **Ru2** amount did not afford better yields (entries 11 and 12). When 0.3 mol % of catalyst was utilized (entries 12 and 13), it was observed that increasing the residence time by 1 min had increased the yield from 73 to 94%. A full conversion was not observed, which probably occurred because of the reversibility of the metathesis reaction within the microreactor tubing as ethylene formed as a byproduct cannot leave out of the reaction mixture.

Next, we proceeded to examine the efficiency of the SnatchCat in the removal of ruthenium contaminant after the reaction with both DCM and EtOAc as shown in Table 2. With reference to previous literature, we performed the postreaction workup in batch for 30 min at room temperature with isocyanide scavengers to remove traces of ruthenium in the product to estimate the timing needed for the elevated temperature in the flow setup.^22,23,33,34^ A simple filtration through a short silica gel was conducted to remove the ruthenium waste from postreaction in flow tubing with SnatchCat. Subsequently, we digested the products obtained and conducted ICP-MS analysis to determine the effectiveness.^29^

**Table 2:**
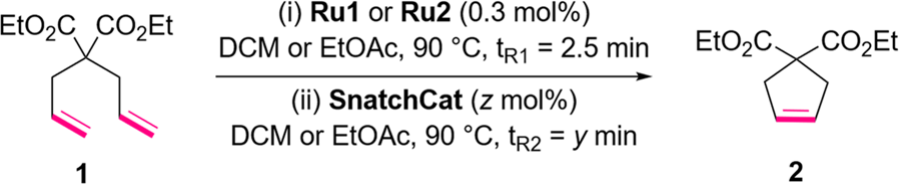
Evaluation of the Time and Concentration of SnatchCat Needed to Reduce the Ruthenium Content Efficiently

entry	solvent	catalyst	*z*(mol %)	*t*_R2_ (*y* min)	yield (%)^a^	ruthenium content in **2** (ppm)^b^
1	DCM	**Ru1**	0.0	0.0	93	21.3
2	DCM	**Ru1**	13.2	2.5	93	2.2
3	DCM	**Ru2**	0.0	0.0	93	1.2
4	DCM	**Ru2**	13.2	2.5	93	0.2
5	DCM	**Ru2**	0.66	5.0	93	0.2
6	DCM	**Ru2**	1.5	5.0	93	0.2
7	EtOAc	**Ru1**	0.0	0.0	98	855.0
8	EtOAc	**Ru1**	13.2	2.5	98	56.3
9	EtOAc	**Ru2**	0.0	0.0	94	15.5
10	EtOAc	**Ru2**	13.2	2.5	94	7.2
11	EtOAc	**Ru2**	13.2	5.0	94	0.6

a Yield determined by ^1^H NMR spectroscopy.

b The content of Ru residue in the product was determined by ICP-MS.

When RCM reaction of **1** was performed in DCM the ruthenium content in purified product was relatively low, ranging from 0.2 ppm (for reaction catalyzed by **Ru2**) to 21.3 ppm (for reaction made in the presence of **Ru1** and without Ru-scavenger) (Table 2, entries 1–6). When the same reaction was performed in EtOAc in the presence of **Ru1** and without SnatchCat, a very high ruthenium content of 855 ppm was detected in the product (Table 2, entry 7). When SnatchCat was introduced for postreaction, the ruthenium content was greatly reduced to 56.3 ppm (entry 8), highlighting the efficiency of SnatchCat metal scavenger. Moreover, the ruthenium content was lowered (7.2 ppm) when **Ru2**, featuring a polar ammonium tag, was used instead of **Ru1** (entry 9). Finally, the utilization of both **Ru2** and SnatchCat allowed for reduction of the heavy metal content to a level acceptable to the pharmaceutical industry (Table 2, entry 10), which demonstrated the efficiency of these reagents used together (polar **Ru2** is sometimes referred as a s*elf-scavenging catalyst*).^29^

**Figure 3:**
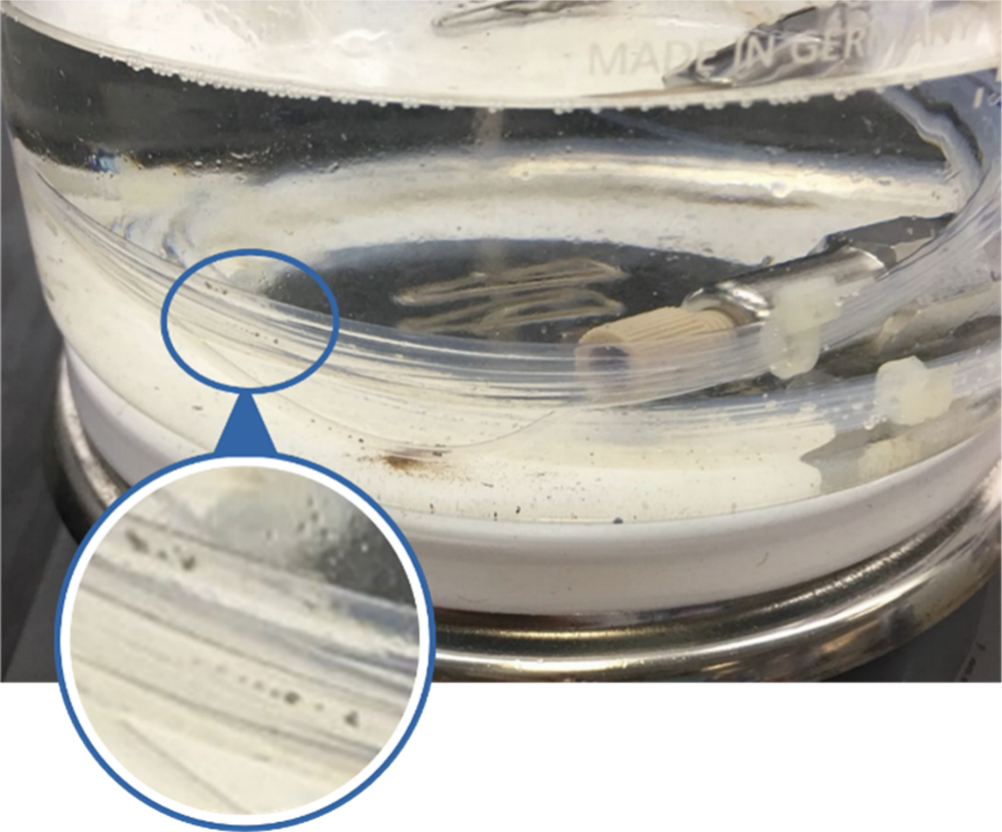
Formation of an undefined ruthenium–SnatchCat insoluble complex in the flow tubing reactor.

Neither the amount of SnatchCat nor the residence time (*t*_R2_) for the postreaction had a significant effect on the ruthenium contamination in product **2** obtained in DCM (Table 2, entries 4–6). However, the situation differs when EtOAc was used as a solvent (Table 2, entries 10–11). Here, by doubling the residence time for the SnatchCat, the residual ruthenium content in the product was significantly reduced from 7.2 to 0.6 ppm (entry 11). We were pleased to see such a low level of Ru-contamination, as EtOAc—a highly polar solvent—was expected to elute them out. Interestingly, the increase of residence time caused the precipitation of undefined residue, being probably a complex of SnatchCat and spent ruthenium catalyst (Figure 3), which could be removed by use of a short plug with silica gel to greatly assist in reducing metal contamination by ruthenium.

### Substrate Scope

After determining the optimal conditions for RCM in continuous flow with both high yield of products and a low ruthenium content, we proceeded to expand the substrate scope. In the present study, we focused on substrates that possess a number of functional groups with Brønsted basic sites that can potentially bind to ruthenium making catalyst traces separation difficult. The concentrations of the substrates were adjusted accordingly to ensure that the formed products are soluble in EtOAc; also, the residence times for most substrates were increased from 2.5 to 5 min due to the increased complexity of the starting materials.

Relacatib is a drug with high potency to inhibit cathepsin K.^71^ Its precursor—allyl(1-methylpent-4-enyl)carbamic acid benzyl ester (**3**)—was tested in the RCM reaction in both flow and batch. In the reaction performed in batch, the seven-membered ring product **4** was obtained in 99% yield. To our delight, we found that almost the same result can be repeated in flow as product **4** was obtained in 96% yield and with very low Ru-content of 0.4 ppm despite the presence of an ester group and a nitrogen atom which may limit the effectiveness of SnatchCat (Table 3, entry 1).^34^ The superheating at 90 °C in flow setup overcame activation energy and reduced the reaction time significantly compared with batch. Compound **6** which can be obtained in RCM of 5-benzylnona-1,8-dien-5-ol (**5**) is a precursor of Halidor—a FDA-approved drug possessing antispasmodic, vasodilator, and platelet aggregation inhibitor properties.^72^ The free OH group present in the substrate caused a significant reduction of the outcome of reaction carried out under classical conditions (only 30% yield was observed). The use of a flow system had a beneficial effect on the reaction and enabled us to obtain the expected product **6** with 95% yield and low ruthenium residue (0.86 ppm) (Table 3, entry 2). Next, two substrates, 2-allyl-2-phenylpent-4-enoicacid (**7**), which is the precursor of Silomat used in asthma treatment,^73^ and *N,N*-diallyl-1-tosylpyrrolidine-2-carboxamide (**9**), which can be used in synthesis of SUAM 1221, a drug known for treatment of psychological diseases,^74^ that were used in flow provided high yields of the desired products **8** and **10** (96 and 97%, respectively) together with low ruthenium content (<2 ppm). In these cases, similar yields for **8** and **10** were found in the reactions performed in batch (Table 3, entries 3 and 4).

**Table 3:**
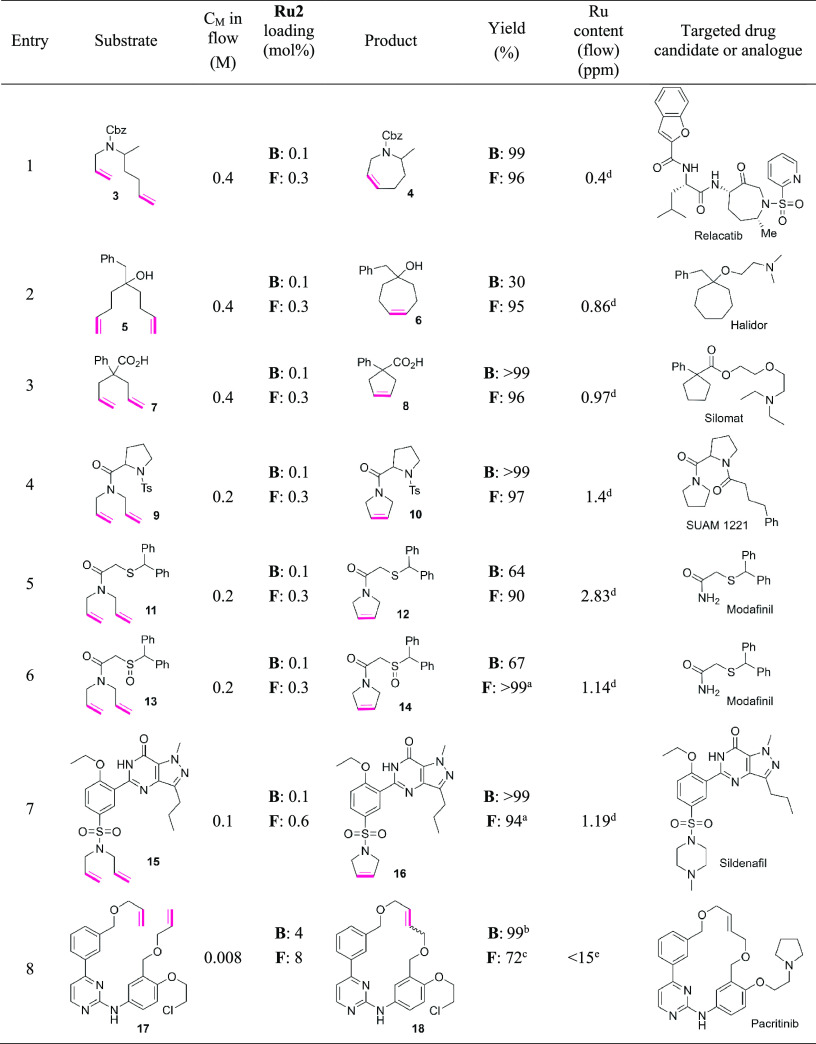
Substrate Scope in Batch and Flow Reactions Catalyzed by StickyCat PF_6_ (**Ru2**)¶

¶ Conditions for reactions in batch (**B**): **Ru2**, EtOAc (0.1 M), 60 °C, 120 min. Yield determined on the basis of GC analysis with durene as an internal standard checked after quenching the reaction mixture with 4.4 equiv of SnatchCat solution. Conditions for reactions in flow (**F**): **Ru2**, EtOAc, 90 °C, *t*_R_ = 5 min. Yield determined on the basis of GC analysis with 1,3,5-trimethoxybenzene as an internal standard.

a Yield determined on the basis of crude ^1^H NMR spectra.

b **Ru2** (added in 4 portions, 1 mol % per hour), TFA (2 equiv), EtOAc (2 mM), 78 °C, 4 h, basic workup in the presence of Na_2_CO_3_. Yield of pure product isolated on column chromatography. (*E*)/(*Z*) = 70:30 determined on the basis of ^1^H NMR.

c **Ru2**, TFA (1 equiv), EtOAc, 90 °C, *t*_R_ = 20 min, basic workup in the presence of Na_2_CO_3_, yield of pure product isolated on column chromatography. (*E*)/(*Z*) = 66:34 determined on the basis of ^1^H NMR.

d Ru-content in product determined by ICP-MS.

e Ru-content in product determined by ICP-OES.

Substrates **11** and **13** are the precursors for an analogue of the Modafinil, the drug which is used to treat sleepiness due to narcolepsy, shift work sleep disorder, or obstructive sleep apnea (OSA).^75^ In batch reactions, the results were moderate, as yields of 64 and 67% (for product **12** and **14**, respectively) were obtained (Table 3, entries 5 and 6). Improved results were obtained when the flow setup was employed; **14** generated by RCM of **13** was obtained in quantitative yield while the reaction of **11** led to **12** in 90% of yield. Furthermore, slightly higher ruthenium contamination was observed for the product bearing the thioether moiety (**12**, 2.83 ppm) compared to the sulfoxide one (**14**, 1.14 ppm). It is worth noting that these two analogues of Modafinil have been previously synthesized in batch in PhMe at 70 °C, in slightly lower yield of 96 and 84% using slimly higher loading of a latent Ru catalyst (0.5 mol %).^76^

Compound **15**, an analogue of Sildenafil—known under the trade named Viagra—a drug used in the treatment of erectile dysfunction and pulmonary arterial hypertension,^77,78^ was tested in both batch and flow reactors with a yield of >99% and 94%, respectively. However, in the flow system, an increase of catalyst loading (from 0.3 to 0.6 mol %) was required to achieve high yield, as 0.3 mol % of catalysts only delivered 30% yield of the product. Nevertheless, the detected ruthenium content was still less than 3 ppm, thus demonstrating the utility of both SnatchCat and **Ru2** in the flow reactor to obtain high product yields with low ruthenium contents. Notably, previously published process for **16** manufacturing at a scale of 33 g that uses a standard batch reactor required up to 1 mol % of catalyst loading to yield **16** in 88% with ruthenium content in product equal 88 ppm.^61^

We further explored the RCM in the preparation of macrocyclic compound **18**, being a precursor of Pacritinib, a drug which has undergone Phase III clinical trials for the treatment of myelofibrosis and lymphoma as a kinase inhibitor.^79^ Because of the presence of Brønsted basic nitrogen in this substrate, the RCM has to be conducted in the presence of a Brønsted acid, such as trifluoroacetic acid (TFA), to avoid deactivation of the Ru catalyst.^80,81^ In batch reactors, diene **17** was first dissolved in EtOAc. TFA was added followed by catalyst **Ru2** (four portions, 1 mol % each, 1 h intervals). When the reaction was completed and the TFA salt of **18** was generated, SnatchCat was added to remove residual ruthenium. The basic workup of the resulting mixture provided **18** in 99% yield. Compared with the batch reaction, the flow reaction (Table 3, entry 8) enabled an increase of substrate concentration as well as omitting the portion-wise addition of catalyst during the reaction in batch. The residence time was also greatly reduced from 4 h to 20 min in the flow reactor. Importantly, the flow system and the use of the metal scavenger have proved to be able to withstand acidic conditions, and the amount of ruthenium content in **18** was below 15 ppm after isolation with a short plug with silica gel.

### Larger-Scale Production

**Figure 4:**
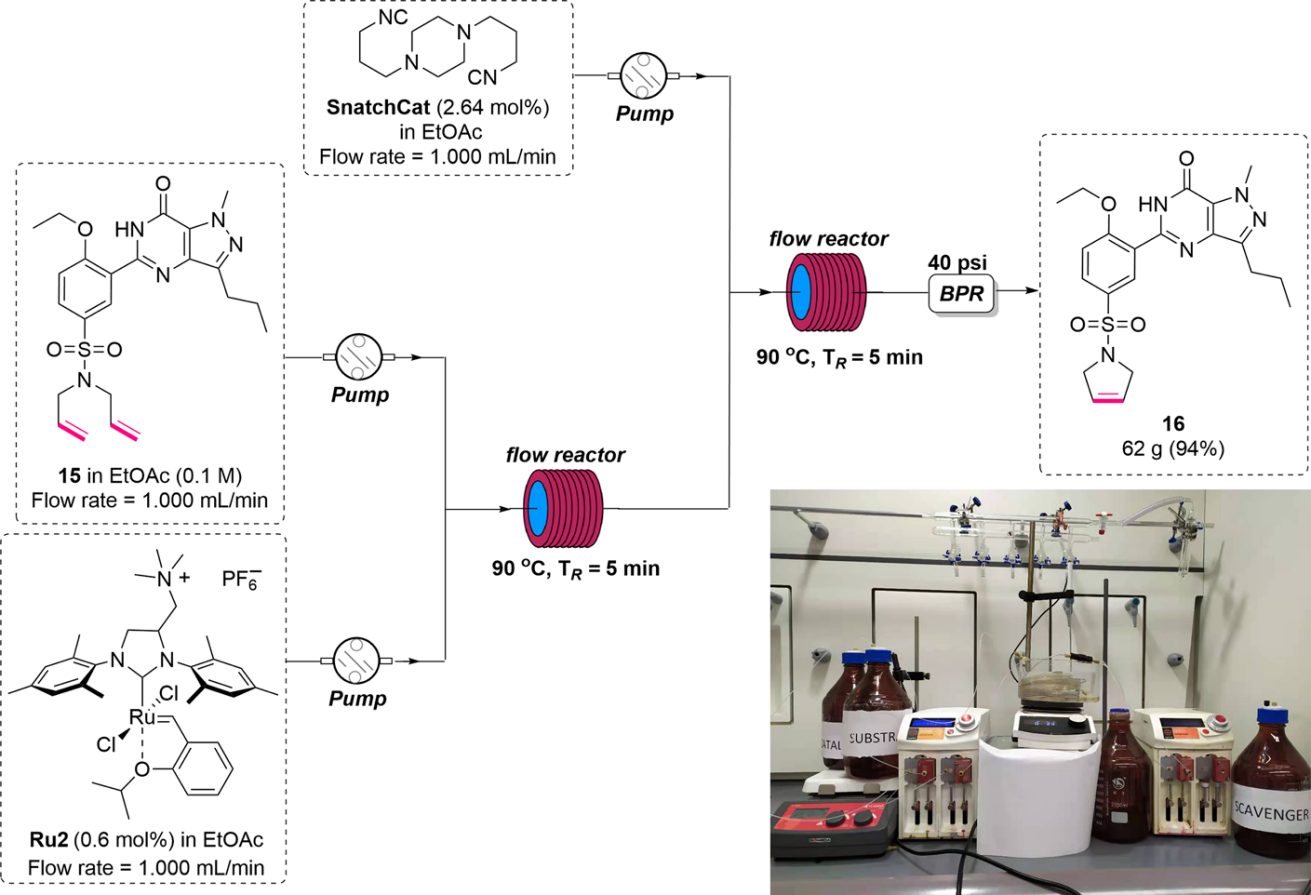
Representative schematic flow setup of 24-h scale production of ring-closing metathesis of **15** in the presence of **Ru2** and SnatchCat.

Encouraged by the results achieved so far, we decided to conduct a larger-scale ring-closing metathesis reaction of **15** in the flow system. After a 24 h process, we were able to produce 62 g of Sildenafil analogue **16** in 94% yield, which is similar to the result obtained in the small-scale reaction. Comparing these two processes (larger scale—Figure 4, small scale—Table 3, entry 7), we enlarged the reactor volume of the microtubing from 2 to 10 mL, which allowed for increasing of the flow rate from 0.2 to 1.0 mL/min, thus augmenting the production rate without changing the concentration and residence time, as shown in Figure 4. Similarly, we expanded the tubing capacity of the scavenging reaction with SnatchCat from 3 to 15 mL to maintain the concentration and residence time for the ruthenium precipitate to form as illustrated in Figure 3. The resulting mixture was collected after 24 h before filtration through a short pad of silica gel using pure ethyl acetate as the solvent. ICP-MS analysis of the obtained crude product indicated that only 0.5 ppm ruthenium was detected. Although the flow system had been upscaled, the reactivity and efficiency of removal of ruthenium was maintained, indicating the suitability of this flow synthesis for industrial applications. It shall be stated that this experiment presents the largest reported scale of intermediate **16** manufacturing. The previous R&D syntheses conducted in Polpharma SA pharmaceutical company were made in 10 g (in DCE, 1 mol % of Ru, 79% yield) and in 33 g (in 4-MeTHP, 1 mol % of Ru, 88% yield) scale in batch automated reactors using mechanical stirring.^61^

## Conclusion

In conclusion, this study presents a simple and environmentally friendly flow setup for ring-closing metathesis that uses a green solvent—ethyl acetate. Using this technique, a number of representative API analogues and precursors containing complex heterocyclic and macrocyclic motifs have been obtained. The application of low loading of ammonium-tagged catalyst and the addition of a ruthenium scavenger—SnatchCat—allowed for the synthesis of these polyfunctional organic compounds in a yield higher than 90% and with the residual ruthenium content below 10 ppm. Importantly, these high conversions have been achieved without active removal of ethylene byproduct (such as tube-in-tube designs^53^), which make the hardware setup much simpler and less costly. In addition, a larger-scale production was performed with small infrastructure space to produce more than 62 g of Sildenafil analogue **16** in 24 h in 94% yield. Importantly, the product collected after a simple filtration through short pad of silica gel contained less than 1 ppm of ruthenium contamination. We believe that with further development, this strategy could be used by the pharmaceutical industry to produce polyfunctional API molecules at larger-scale production and with lower environmental impact, as compared to currently implemented manufacturing methods.

## Supporting Information Available

Experimental details, copies of NMR spectra (PDF)
