# Supplementary material for: Olefin Metathesis in Continuous Flow Reactor Employing Polar Ruthenium Catalyst and Soluble Metal Scavenger for Instant Purification of Products of Pharmaceutical Interest
Source: ACS Sustain Chem Eng. 2021 Nov 22;9(48):16450–8. doi: 10.1021/acssuschemeng.1c06522 (PMC8655794; doi:10.1021/acssuschemeng.1c06522)
Supplement: Supplementary file 1 — sc1c06522_si_001.pdf [file sc1c06522_si_001.pdf]

SUPPORTING INFORMATION

Olefin Metathesis in Continuous Flow Reactor Employing Polar Ruthenium Catalyst and Soluble Metal Scavenger for Instant Purification of Products of Pharmaceutical Interest

Ren Wei Toh,[a,‡] Michał Patrzałek,[b,‡] Tomasz Nienałtowski,[b,c] Jakub Piątkowski,[b] Anna Kajetanowicz,[b,] Jie Wu,[a,*] Karol Grela[b,*]*

[a]Department of Chemistry, National University of Singapore, 3 Science Drive 3, Singapore 117543, Singapore

[b]Biological and Chemical Research Centre, Faculty of Chemistry, University of Warsaw, Żwirki i Wigury 101, 02-089 Warsaw, Poland

[c]Pharmaceutical Works Polpharma SA, Pelplińska 19, 83-200 Starogard Gdański, Poland

E-mails of corresponding authors:
prof.grela@gmail.com (K. Grela);
chmjie@u.nus.edu (J. Wu);
a.kajetanowicz@uw.edu.pl (A. Kajetanowicz)

Number of pages: 24
Number of tables: 1
Number of figures: 23
Number of schemes: 4

Table of Contents

Materials and Methods 3
Reactions in Batch 4
General Procedure for RCM (Ring Closing Metathesis) Reactions in Batch 4
Procedure for RCM Reaction of Pacritinib Precursor in Batch 6
Reactions in Flow 7
Flow System Details and Equipment 7
General Procedure for RCM (Ring Closing Metathesis) Reactions in Flow in Small Scale 8
Procedure for RCM Reaction of Sildenafil Derivative in Flow in Larger Scale 10
Analytical Data 12
GC Quantification Details 14
ICP-OES Measurement Details 14
^{1}H and ^{13}C NMR Spectra 15
References 24

Materials and Methods

General Remarks: unless otherwise noted, all materials were purchased from commercial suppliers and used as received. All reactions requiring exclusion of oxygen and moisture were carried out in oven-dried glassware with dry solvents (purified using MBraun SPS) under a dry and oxygen-free atmosphere using Schlenk technique. The bottles with ruthenium catalysts were stored under argon atmosphere, but no special precautions were taken to avoid air or moisture exposure in the moment of extracting the catalysts from the bottles.

Analytical Reagent (AR) Grade Ethyl Acetate (EtOAc) or Dichloromethane (DCM) was used for the batch and flow reactions. The solvent was dried over 4Å molecular sieves (dried in the oven at 100 °C overnight) and bubbled with argon gas for at least 15 minutes.

Nuclear Magnetic Resonance (NMR): NMR (^{1}H and ^{13}C) spectra were recorded on *Agilent* Mercury 400 MHz, *Bruker* AV-III400 400 MHz, or AMX500 500 MHz spectrometers at ambient temperature with $CDCl_3$ and CD_2Cl_2 used as a solvent purchased from Sigma-Aldrich or Eurisotop which was stored over molecular sieves and silver foil. Chemical shifts (δ) are given in parts per million (ppm) downfield from tetramethylsilane with a residual protio solvent signal used as a reference: $CDCl_3$ (δ_H = 7.26 ppm, δ_C = 77.16 ppm), CD_2Cl_2 (δ_H = 5.32 ppm, δ_C = 54.00 ppm). Coupling constants (*J*) are reported in hertz (Hz) and refer to *H*,*H*-coupling. The following abbreviations are used in order to indicate the multiplicity of the signal: s (singlet), d (doublet), t (triplet), q (quartet), quin (quintet), sext (sextet), hept (heptet), dd (doublet of doublet), dt (doublet of triplet), ddd (doublet of doublet of doublet) etc., bs (broad signal), m (multiplet). The obtained data was processed using MestReNova software.

Gas Chromatography (GC): GC measurements conducted at the Biological and Chemical Research Centre were carried out using *PerkinElmer Clarus 580* with FID detector employing InertCap 5MS-Sil Capillary Column (inner diameter 0.25 mm, length 30 m, df 0.50 μm) and helium as a carrier gas. Analyses performed at the National University of Singapore (under the supervision of Associate Professor Ge Shaozhong) were carried out using *Shimadzu GC-2030AF-1095*.

Column Chromatography (CC): CC was performed using *Merck* Millipore silica gel (60, particle size 0.043 – 0.063 nm) or SANPONT silica gel 60 (63-200 μm) with EtOAc/*n*-hexane system as eluent, unless otherwise stated.

Thin Layer Chromatography (TLC): TLC was performed using *Merck* Silica Gel 60 F_{254} precoated aluminum sheets (0.25 mm thickness). Substances were visualized using UV-light (254 or 365 nm) or by stains: $KMnO_4/K_2CO_3/NaOH$ (aqueous solution) or anisaldehyde/H_2SO_4 (ethanolic solution).

Inductively Coupled Plasma Optical Emission Spectrometry (ICP-OES): ICP-OES results were obtained at EA analytical laboratory at the National University of Singapore using *Perkin Elmer Avio 500* spectrometer.

Reactions in Batch

General Procedure for RCM (Ring Closing Metathesis) Reactions in Batch

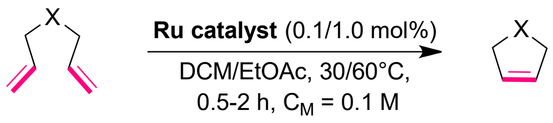

Scheme S1. General scheme for RCM reactions in batch.

Reactions were performed in dry solvents (DCM or EtOAc) under argon atmosphere. Stock solution of catalyst was prepared by dissolving 0.02 mmol of the appropriate catalyst (**Ru1** or **Ru2**) in 1 mL of DCM or EtOAc (C_M = 0.02 M). 0.1 M substrate solution (3 mL, 0.3 mmol, 1 equiv.) was placed in an oven-dried vial followed by the addition of 0.02 M solution of the catalyst (15/150 µL, 0.0003/0.0030 mmol, 0.1/1.0 mol%). The reaction mixture was stirred at the given temperature (30/60 °C) and then, the solution of SnatchCat (0.045 M in DCM) was added (0.44/4.4 mol% respectively for 0.1/1.0 mol% of the ruthenium catalyst). The resulting mixture was stirred for 30-120 min (200 rpm). The product was purified using column chromatography (stationary phase: SiO_2, eluent: reaction solvent). Yields for all substrates but **15** were calculated based on GC results using durene (1,2,4,5-tetramethylbenzene) as an internal standard or NMR experiments (for substrate **15**).

Note: Compound **16** precipitated from the reaction mixture as colorless crystals when the reaction is carried out in EtOAc.

Table S1. Results of RCM reaction performed in batch.

Substrate	Catalyst	Solvent	Loading [mol%]	Product	Yield [%] (30 °C)	Yield [%] (60 °C)
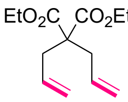 **1**	**Ru1**	DCM	1.0	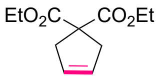 **2**	99	-
			0.1		83	-
		EtOAc	1.0		94	99
			0.1		30	31
	**Ru2**	DCM	1.0		99	-
			0.1		81	-
		EtOAc	1.0		96	99
			0.1		33	33
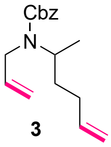 **3**	**Ru1**	DCM	1.0	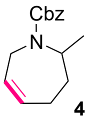 **4**	99	-
			0.1		99	-
		EtOAc	1.0		99	99
			0.1		99	99
	**Ru2**	DCM	1.0		99	-
			0.1		99	-
		EtOAc	1.0		99	99
			0.1		99	99
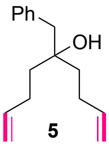 **5**	**Ru1**	DCM	1.0	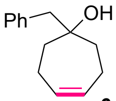 **6**	99	-
			0.1		7	-
		EtOAc	1.0		94	99
			0.1		30	31

	Ru2	DCM	1.0		73	-
			0.1		1	-
		EtOAc	1.0		96	99
			0.1		33	33
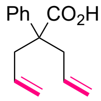 **7**	Ru1	DCM	1.0	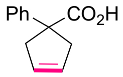 **8**	99	-
			0.1		99	-
		EtOAc	1.0		99	99
			0.1		99	99
	Ru2	DCM	1.0		99	-
			0.1		99	-
		EtOAc	1.0		99	99
			0.1		99	99
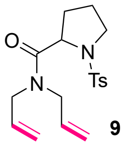 **9**	Ru1	DCM	1.0	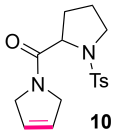 **10**	99	-
			0.1		99	-
		EtOAc	1.0		99	99
			0.1		99	99
	Ru2	DCM	1.0		99	-
			0.1		99	-
		EtOAc	1.0		99	99
			0.1		99	99
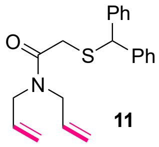 **11**	Ru1	DCM	1.0	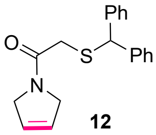 **12**	99	-
			0.1		66	-
		EtOAc	1.0		96	99
			0.1		57	91
	Ru2	DCM	1.0		99	-
			0.1		18	-
		EtOAc	1.0		97	99
			0.1		64	70
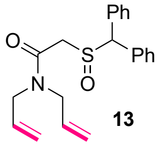 **13**	Ru1	DCM	1.0	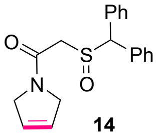 **14**	99	-
			0.1		69	-
		EtOAc	1.0		99	99
			0.1		61	99
	Ru2	DCM	1.0		99	-
			0.1		56	-
		EtOAc	1.0		99	99
			0.1		67	99
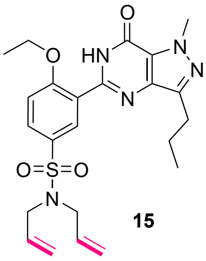 **15**	Ru1	DCM	1.0	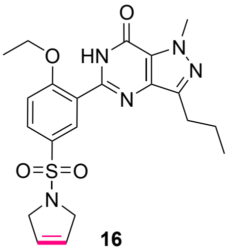 **16**	99	-
			0.1		99	-
		EtOAc	1.0		99	99
			0.1		99	99
	Ru2	DCM	1.0		99	-
			0.1		99	-
		EtOAc	1.0		99	99
			0.1		99	99

Conditions: substrate concentration - 0.1 M; reaction time for: EtOAc (1 mol% of catalyst) - 30 min, DCM (1 mol% of catalyst) - 60 min, EtOAc/DCM (0.1 mol% of catalyst) - 120 min. Yields (**1**, **3**, **5**, **7**, **9**, **11**, and **13**) were calculated using GC with 1,2,4,5-tetramethylbenzene (durene) as internal standard or ^{1}H NMR in case of **15**.

Procedure for RCM Reaction of Pacritinib Precursor in Batch

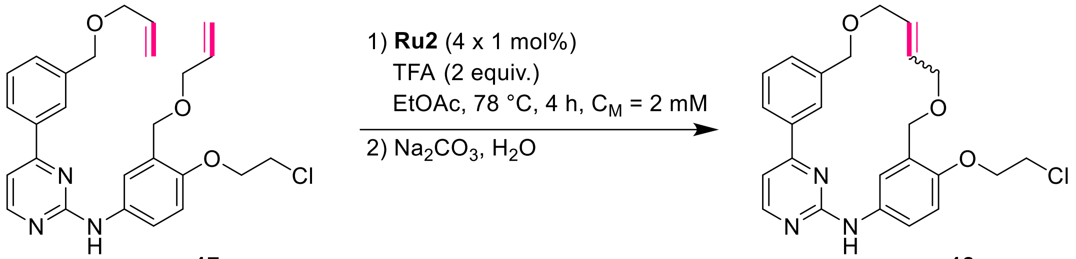

Scheme S2. Scheme of RCM reaction of Pacritinib precursor in batch.

Two-necked round-bottom flask was charged with 50 mL of EtOAc. Then, the precursor of Pacritinib **17** (50 mg, 0.1 mmol, 1 equiv.) was added (C_M = 2 mM). Afterwards, trifluoroacetic acid (24.7 mg, 0.2 mmol, 2 equiv.) and 0.1 mL of **Ru2** solution in DCM (9 mg in 1 mL) were added to the reaction mixture (0.9 mg, 0.001 mmol, 1 mol%). The reaction was carried out at 78 °C. After each hour, next portion of the catalyst was added (1 mol%). A total of 4 mol% of the catalyst (4 portions - 0.1 mL each) was added. Next, SnatchCat (0.0176 mmol, 17.6 mol% *vs* substrate, 4.4 equiv. *vs* the catalyst) solution was added, solvents were evaporated and EtOAc/Na_2CO_3 extraction was performed (3 x 30 mL). Then, the crude product, after drying over Na_2SO_4, was purified using column chromatography (stationary phase: SiO_2, eluent: EtOAc/*n*-hexane 15:85 to 20:80 v/v) to obtain 45.5 mg (97%) of the product **18** (*E*/*Z* = 70:30). The product forms yellow feathers.

Notes:

- after basic extraction the product does not contain TFA anion – it appears as free amine form (based on ^{19}F NMR)
- *E*/*Z* ratio was found by calculations based on NMR signals. Two appropriate signals (one from *E*-isomer and second from *Z*-isomer) were found and integrated (7.81 ppm – *E*-form, 7.76 ppm – *Z*-form). The integral sum was set at the value of 100.

Reactions in Flow

Flow System Details and Equipment

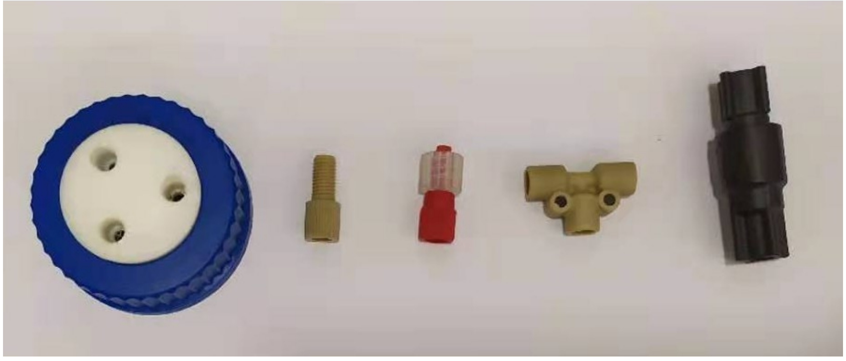

Figure S1. Flow system parts. From left to right: custom-made blue caps; 1/8 or 1/16 HPFA nut part; needle connector used for extracting reaction mixture into KDS stainless steel syringe; T-connector; back pressure regulator, 40 psi. Except for the blue caps, the remaining items can be purchased from *IDEX Health & Science LLC.*

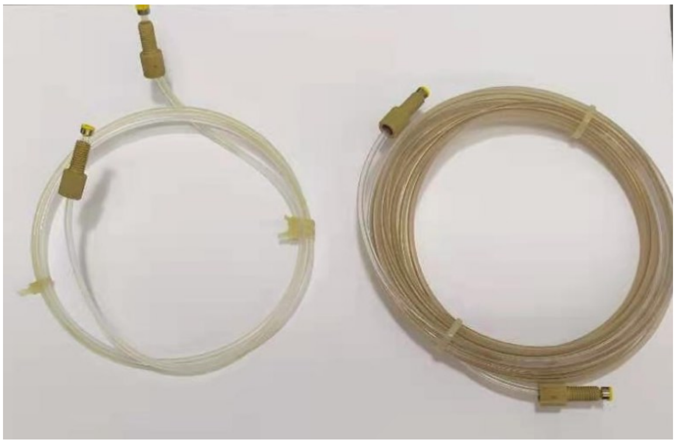

Figure S2. HPFA tubing. Details: 1/8, O.D. 1/16", I.D. 0.062"; left, small scale reaction, 2 mL; right, large scale reaction, 10 mL). Items can be purchased from *IDEX Health & Science LLC*.

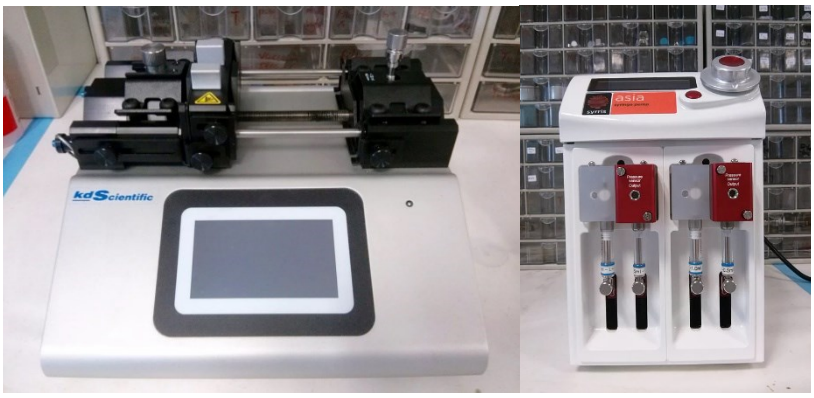

Figure S3. Pumps used along with flow set-up. The left one for small scale reactions (KDS syringe pump) and the right one for large scale reactions (*Asia* pump).

General Procedure for RCM (Ring Closing Metathesis) Reactions in Flow in Small Scale

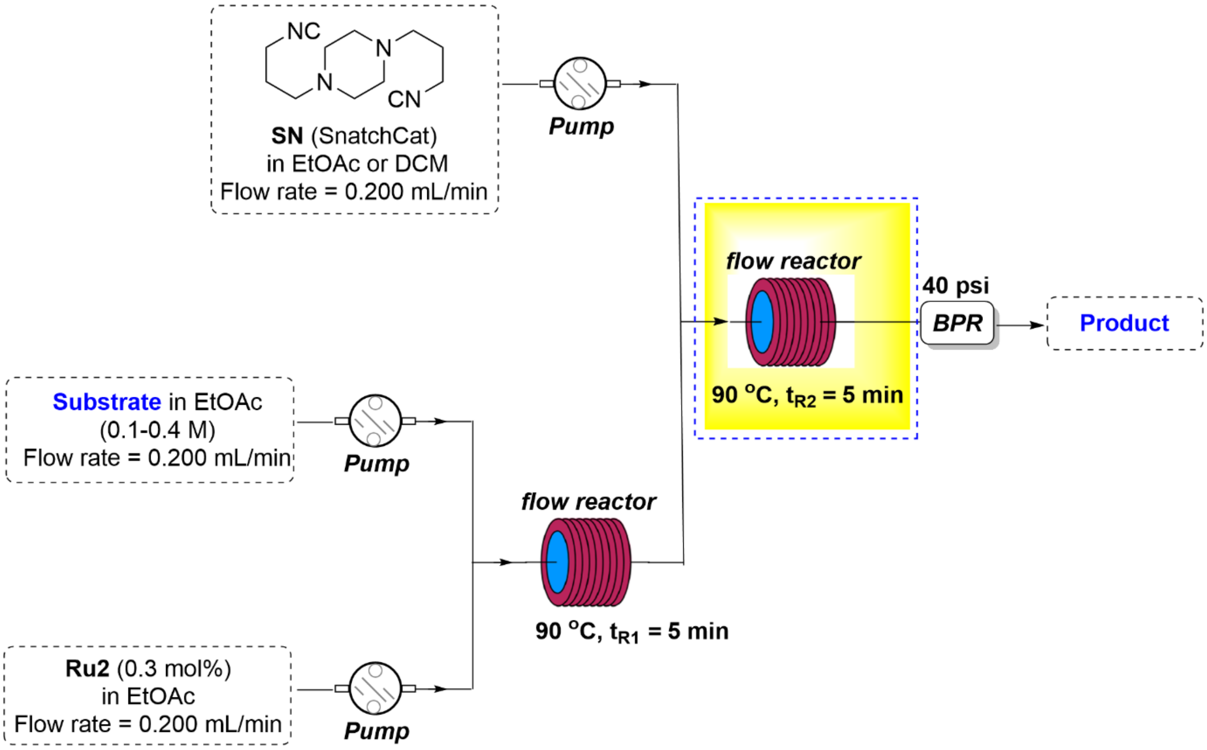

Scheme S3. General flow set-up diagram for testing substrates in small scale with varying concentration of the substrates depending on the solubility.

Substrate (2.4 mmol, 1 equiv.) and the catalyst **Ru2** (6.1 mg, 0.0072 mmol, 0.3 mol%) were weighed separately, put into two different 20 mL vials (preheated in the oven for at least 30 min), and covered with rubber septum caps tightly. Atmosphere exchange was done employing argon (as an inert gas) using vacuum pumps (three cycles were performed before attaching balloons filled with argon to each of the vials). Then, 8 mL of dried and argon-bubbled EtOAc was transferred into the vials *via* a disposable syringe and sonication was done to ensure that all the catalyst and substrate were fully dissolved. The final concentration before mixing in the T-connector for the substrate was 0.4 M. Concentration of the substrates were varied based on the solubility in EtOAc.

Using an 8 mL KDS stainless syringe attached with a needle connector (**Figure S1**), the substrate solution was extracted from the 20 mL vial and connected to a KDS syringe pump. Another 8 mL KDS stainless syringe was filled with the **Ru2** solution mixture and attached to the same KDS syringe pump. Meanwhile, SnatchCat scavenger (6.1 mg, 0.030 mmol, 1.32 mol%, 4.4 equiv. *vs* the catalyst) was dissolved in 5 mL of EtOAc and transferred into an 8 mL KDS stainless syringe. Likewise, the syringe was attached to a KDS syringe pump. The flow set-up is as shown in **Scheme S3** with the tubing soaked in an oil bath (**Figure S4**) to provide heating for the reaction. After 2 residence timing (t_R) of 20 minutes, the product was collected at the end of the silica packed-bed and concentrated under vacuum before further analysis using GC, NMR or ICP-MS were performed.

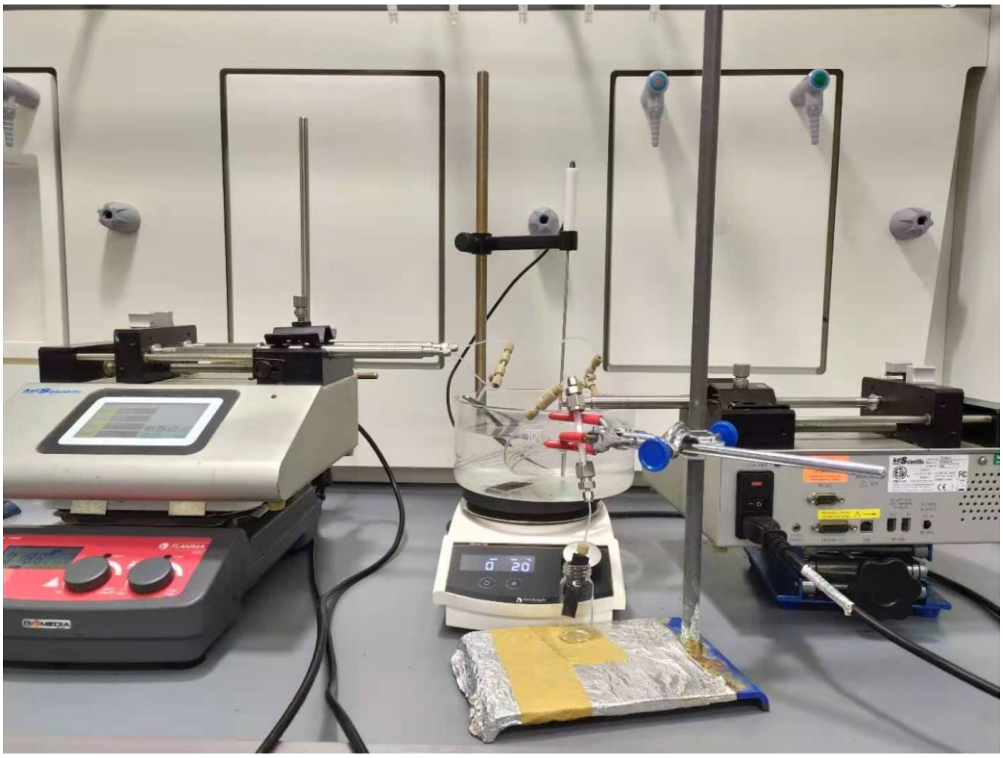

Figure S4. General flow set-up for testing substrates in small scale. The pump on the left was used for pumping substrates and catalyst, while the pump on the right was used to pump SnatchCat for the work-up. At the end of the reaction, the residues were filtered through the silica gel packed bed and collected in the 20 mL glass vial.

Procedure for RCM Reaction of Pacritinib Precursor in Flow

The precursor of Pacritinib **17** (30 mg, 0.064 mmol, 1 equiv.) and catalyst **Ru2** (4.3 mg, 0.005 mmol, 8.0 mol%) were weighed separately, put into two different 20 mL vials (preheated in the oven for at least 30 min), and covered with rubber septum caps tightly. Atmosphere exchange was done employing argon (as inert gas) using vacuum pumps (three cycles were performed before attaching balloons filled with argon to each of the vials). Then, 8 mL of dried and argon-bubbled EtOAc was transferred into the vials *via* a syringe and sonication was done to ensure that all the catalyst and substrate were fully dissolved. Afterwards, 10 μL of trifluoroacetic acid (15 mg, 0.135 mmol, 2.1 equiv.) was added to the Pacritinib precursor solution in order to acidify the free amine groups in the starting material *via* a micro-syringe.

Using an 8 mL KDS stainless syringe attached with a needle connector (**Figure S1**), the acidified Pacritinib solution mixture was extracted from the 20 mL vial and connected to a KDS syringe pump. Another 8 mL KDS stainless syringe was filled with the **Ru2** solution mixture and attached to the same KDS syringe pump. Meanwhile, solid SnatchCat (5.0 mg, 0.023 mmol, 35.2 mol%, 4.4 equiv. *vs* the catalyst) was dissolved in 8 mL of untreated EtOAc and extracted into an 8 mL KDS stainless syringe. Likewise, the syringe was attached to a KDS syringe pump. The flow set-up was as shown in **Scheme S3** with the tubing soaked in an oil bath at 90 °C to provide heating for the reaction. After 2 residence timing (t_R) of 40 minutes, the product was collected at the end of the silica packed bed and concentrated under vacuum. Concentrated sodium carbonate

solution was used to neutralize the product *via* liquid-liquid extraction. The organic layer was concentrated and purified using flash column chromatography (stationary phase: SiO_2, eluent: EtOAc/*n*-hexane 20:80 v/v) to obtain the desired RCM product **18** (72%, *E*/*Z* = 66:34). Further analysis was performed using NMR and ICP-OES (<15 ppm of Ru).

Procedure for RCM Reaction of Sildenafil Derivative in Flow in Larger Scale

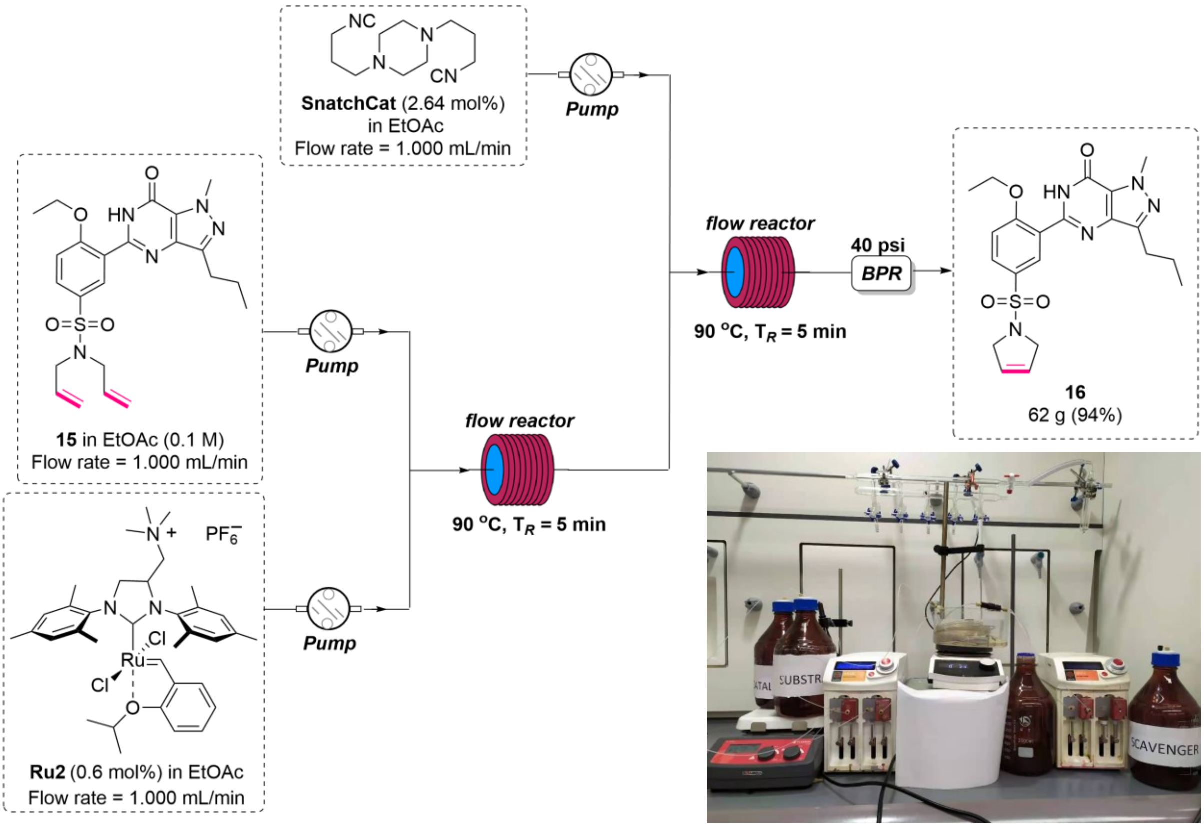

Scheme S4. Flow set-up diagram for the synthesis of Sildenafil derivative in a 24-hour flow system.

Sildenafil derivative **15** (72.84 g, 150 mmol, 1 equiv.) and catalyst **Ru2** (759.6 mg, 0.9 mmol, 0.3 mol%) were weighed separately, put into two different 2.5 L blue capped bottles (preheated in the oven for at least 30 min), and tightened with the blue cap shown in **Figure S1**. The starting materials were purged with argon gas for 10 minutes to remove any residual air in the bottle. Subsequently, 1.5 L of dried and argon-bubbled EtOAc was transferred into the bottles and sonicated until all the catalyst and substrate were fully dissolved into the solvent.

The two large bottles were connected to an *Asia* pump which was connected to the reaction tubing. Meanwhile, SnatchCat scavenger (871.2 mg, 3.96 mmol, 1.32 mol%, 4.4 equiv. *vs* the catalyst) was dissolved in 1.5 L of EtOAc and connected to another *Asia* pump (shown in **Scheme S4** and **Figure S5**). The flow set-up was illustrated in **Scheme S4** with the tubing soaked in an oil bath at 90 °C to provide heating for the reaction. After 2 residence timing (t_R) of 20 minutes, the product was collected at the end. The post-reaction mixture was being collected for 24 hours and then purified using flash column chromatography (stationary phase: 60 g of SiO_2, eluent: EtOAc). The product solution was concentrated under vacuum to furnish product **16** (62 g, 94%). The product was analyzed using NMR and ICP-MS (0.51 ppm of Ru).

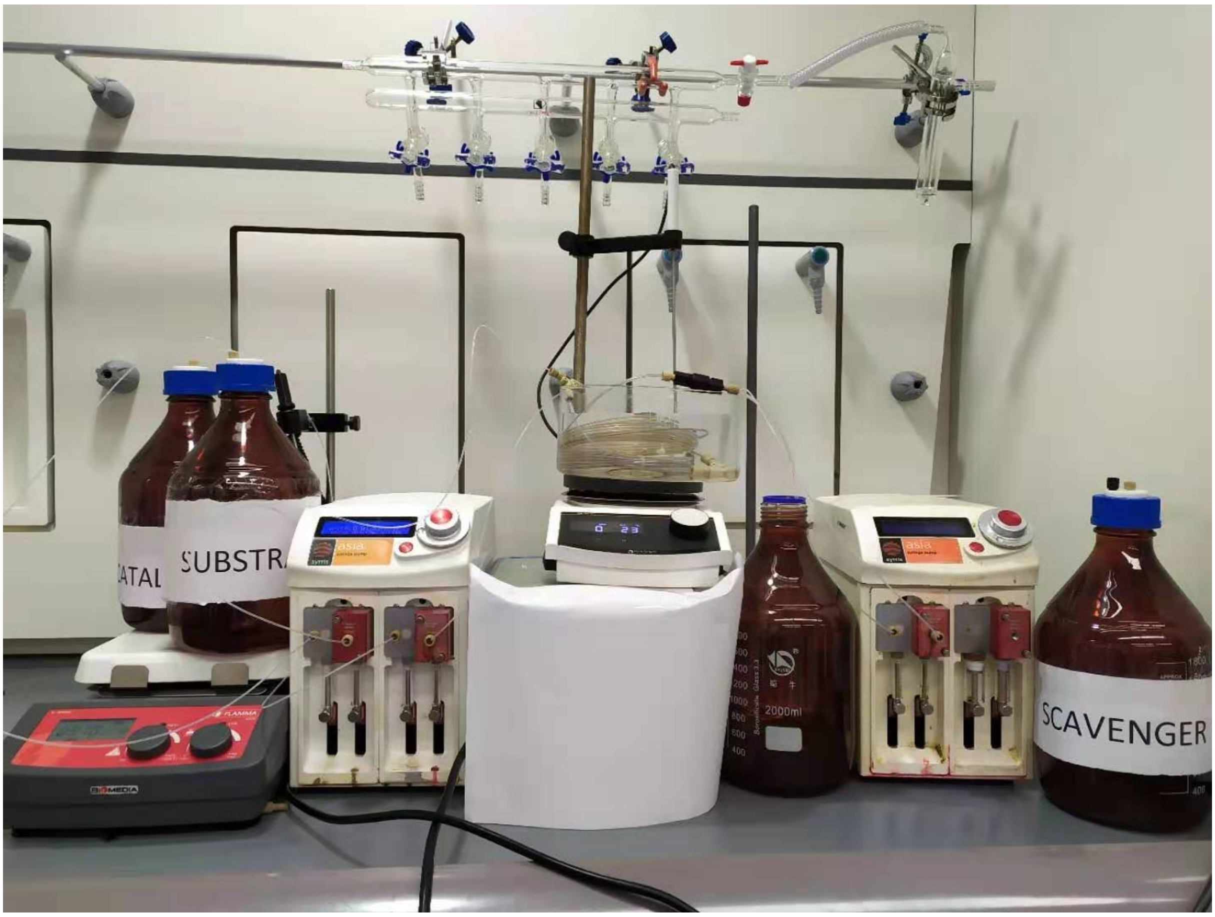

Figure S5. Actual flow set-up for the synthesis of Sildenafil derivative **16** in a 24-hour flow system with two *Asia* pumps.

Analytical Data

Diethyl cyclopent-3-ene-1,1-dicarboxylate (**2**)[1]

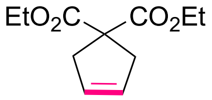

Colorless liquid purified using column chromatography (stationary phase: SiO_2, eluent: EtOAc/*n*-hexane 10:90 v/v).

^{1}H NMR (400 MHz, $CDCl_3$) δ: 5.61 (s, 2H), 4.20 (q, *J* = 7.1 Hz, 4H), 3.01 (s, 4H), 1.25 (t, *J* = 7.1 Hz, 6H).

^{13}C NMR (101 MHz, $CDCl_3$) δ: 172.2, 127.8, 61.5, 58.8, 40.8, 14.0.

Benzyl 2-methyl-2,3,4,7-tetrahydro-1*H*-azepine-1-carboxylate (**4**)[2]

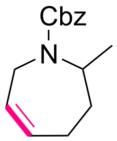

Colorless liquid purified using column chromatography (stationary phase: SiO_2, eluent: EtOAc/*n*-hexane 5:95 v/v).

^{1}H NMR (400 MHz, $CDCl_3$) δ: 7.39–7.28 (m, 5H), 5.68 (dt, *J* = 9.6, 5.6 Hz, 2H), 5.20–5.09 (m, 2H), 4.47–4.06 (m, 2H), 3.65–3.56 (m, 1H), 2.27–2.07 (m, 2H), 1.93–1.66 (m, 2H), 1.14 (dd, *J* = 6.4, 3.6 Hz, 3H).

^{13}C NMR (101 MHz, $CDCl_3$) δ: 156.0, 156.0, 131.7, 131.4, 128.4, 128.3, 127.9, 127.7, 127.7, 127.4, 66.9, 66.7, 52.5, 52.3, 39.4, 39.1, 34.0, 33.9, 27.2, 26.9, 19.6, 19.2.

1-Benzylcyklohept-4-en-1-ol (**6**)[3]

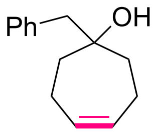

Colorless liquid purified using column chromatography (stationary phase: SiO_2, eluent: EtOAc/*n*-hexane 20:80 v/v).

^{1}H NMR (400 MHz, $CDCl_3$) δ: 7.38 – 7.31 (m, 2H), 7.29 – 7.19 (m, 3H), 5.76 (qt, *J* = 10.6, 4.5 Hz, 2H), 2.92 – 2.73 (m, 2H), 2.36 – 2.00 (m, 2H), 2.09 – 1.97 (m, 2H), 1.71 – 1.67 (m, 2H), 1.37 (d, *J* = 12.5 Hz, 1H).

^{13}C NMR (101 MHz, $CDCl_3$) δ: 137.2, 131.6, 130.7, 128.2, 126.5, 74.9, 48.5, 38.5, 23.0.

1-Phenylcyclopent-3-ene-1-carboxylic acid (**8**)[4]

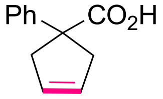

Colorless solid purified using column chromatography (stationary phase: SiO_2, eluent: EtOAc/*n*-hexane 60:40 v/v).

^{1}H NMR (400 MHz, $CDCl_3$) δ: 7.51 – 7.20 (m, 5H), 5.78 (d, *J* = 7.9 Hz, 2H), 3.56 – 3.37 (m, 2H), 2.87 – 2.71 (m, 2H).

^{13}C NMR (101 MHz, $CDCl_3$) δ: 182.6, 142.7, 129.2, 128.5, 127.1, 127.0, 58.4, 42.3.

1-(Tosylpropyl)-2,5-dihydro-1*H*-pyrrole (**10**)[5]

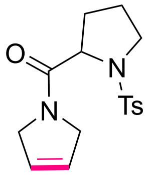

Colorless crystals purified using column chromatography (stationary phase: SiO_2, eluent: EtOAc/*n*-hexane 20:80 v/v).

^{1}H NMR (400 MHz, $CDCl_3$) δ: 7.80 – 7.70 (m, 2H), 7.32 – 7.24 (m, 2H), 5.90 – 5.76 (m, 2H), 4.65 – 4.52 (m, 2H), 4.35 – 4.10 (m, 3H), 3.43 (qdd, *J* = 9.6, 7.3, 6.1 Hz, 2H), 2.40 (s, 3H), 2.18 – 1.89 (m, 3H), 1.85 – 1.71 (m, 1H).

^{13}C NMR (101 MHz, $CDCl_3$) δ: 169.6, 143.4, 135.8, 129.5, 127.5, 127.5, 125.9, 125.9, 125.0, 59.1, 53.4, 53.1, 48.3, 30.3, 24.9, 24.9, 21.5.

2-(Benzhydrylthio)-1-(2,5-dihydro-1*H*-pyrrol-1-yl)ethan-1-one (**12**)[6]

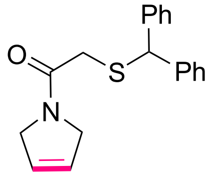

Colorless crystals purified using column chromatography (stationary phase: SiO_2, eluent: EtOAc/*n*-hexane 25:75 v/v).

^{1}H NMR (400 MHz, $CDCl_3$) δ: 7.51 – 7.42 (m, 4H), 7.36 – 7.18 (m, 6H), 5.88 – 5.79 (m, 1H), 5.77 – 5.69 (m, 1H), 5.47 (s, 1H), 4.24 – 4.11 (m, 4H), 3.11 (s, 2H).

^{13}C NMR (101 MHz, $CDCl_3$) δ: 167.2, 140.7, 128.6, 128.5, 127.3, 126.1, 124.9, 53.5, 53.4, 53.2, 34.0.

2-(Benzhydrylsulfinyl)-1-(2,5-dihydro-1*H*-pyrrol-1-yl)ethan-1-one (**14**)[6]

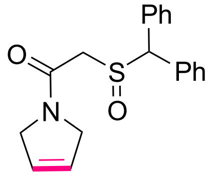

Colorless crystals purified using column chromatography (stationary phase: SiO_2, eluent: EtOAc/*n*-hexane 40:60 v/v).

^{1}H NMR (400 MHz, $CDCl_3$) δ: 7.58 – 7.49 (m, 4H), 7.35 (dq, *J* = 20.1, 7.1 Hz, 6H), 5.80 (dq, *J* = 6.5, 2.1 Hz, 1H), 5.68 (dt, *J* = 6.5, 2.1 Hz, 1H), 5.44 (s, 1H), 4.28 – 4.13 (m, 3H), 4.01 (ddq, *J* = 14.0, 5.0, 2.3 Hz, 1H), 3.48 (d, *J* = 2.5 Hz, 2H).

^{13}C NMR (101 MHz, $CDCl_3$) δ: 162.9, 136.0, 133.7, 130.0, 129.1, 129.0, 128.7, 128.5, 128.2, 125.8, 124.8, 77.5, 77.4, 77.2, 76.9, 69.7, 53.9, 53.7, 53.1.

5-(5-((2,5-Dihydro-1*H*-pyrrol-1-yl)sulfonyl)-2-ethoxyphenyl)-1-methyl-3-propyl-1,6-dihydro-7*H*-pyrazolo[4,3-*d*]pyrimidin-7-one (**16**)[7]

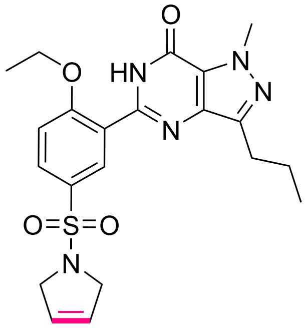

Colorless crystals purified using column chromatography (stationary phase: SiO_2, eluent: EtOAc/*n*-hexane 60:40 v/v).

^{1}H NMR (400 MHz, $CDCl_3$) δ: 10.88 (s, 1H), 8.80 (d, *J* = 2.4 Hz, 1H), 7.85 (dd, *J* = 8.8, 2.4 Hz, 1H), 7.12 (d, *J* = 8.8 Hz, 1H), 5.66 (s, 2H), 4.33 (q, *J* = 7.0 Hz, 2H), 4.22 (s, 3H), 4.14 (s, 4H), 2.89 (dd, *J* = 8.0, 7.1 Hz, 2H), 1.89 – 1.76 (m, 2H), 1.59 (t, *J* = 7.0 Hz, 3H), 0.99 (t, *J* = 7.4 Hz, 3H).

^{13}C NMR (101 MHz, $CDCl_3$) δ: 159.2, 153.6, 146.9, 146.6, 138.3, 131.3, 130.7, 130.5, 125.5, 124.4, 121.1, 113.2, 66.0, 55.0, 38.2, 27.7, 22.3, 14.5, 14.0.

4^4-(2-Chloroethoxy)-6,11-dioxa-3-aza-2(4,2)-pyrimidina-1,4(1,3)-dibenzenacyclododecaphan-8-ene (**18**)[8]

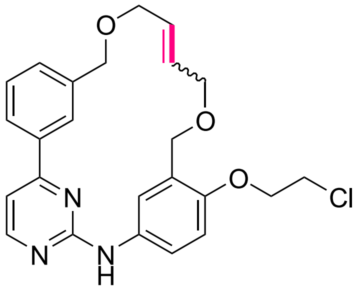

Yellow solid purified using column chromatography (stationary phase: SiO_2, eluent: EtOAc/*n*-hexane 15:85 to 20:80 v/v).

^{1}H NMR (400 MHz, CD_2Cl_2) δ: 8.64 (d, *J* = 1.7 Hz, 1H), 8.51 – 8.38 (m, 1H), 8.26 (d, *J* = 1.8 Hz, 1H), 7.84 (dt, *J* = 7.7, 1.6 Hz, 1H), 7.61 – 7.57 (m, 1H), 7.53 – 7.46 (m, 1H), 7.29 (br, 1H), 7.20 (d, *J* = 5.0 Hz, 1H), 6.90 – 6.86 (m, 2H), 5.91 – 5.66 (m, 2H), 4.68 – 4.56 (m, 4H), 4.29 – 4.24 (m, 2H), 4.13 (d, *J* = 5.8 Hz, 2H), 4.04 (dd, *J* = 5.4, 1.0 Hz, 2H), 3.86 (td, *J* = 5.9, 0.9 Hz, 2H).

^{13}C NMR (101 MHz, CD_2Cl_2) δ: 164.5, 160.7, 159.3, 151.8, 139.4, 137.5, 134.4, 132.2, 131.3, 130.4, 129.3, 127.8, 126.6, 121.7, 119.5, 114.0, 108.4, 70.5, 70.0, 69.7, 68.0, 65.9, 43.0.

GC Quantification Details

Measurements were carried out at National University of Singapore. Sample preparation was done by measuring 5 mg of the starting material (substrates) into GC vials. A standard stock solution of 56.0 mg of 1,3,5-trimethoxybenzene was measured and added with 10 mL of EtOAc as solvent. For each substrate, 1.5 mL of the stock solution was added to it and measured with a GC using temperature programming. The K value of the ratio between the internal standard and substrate was obtained. In addition, the retention time of the substrate was obtained. Subsequently, 5 mg of product sample was measured and added with 1.5 mL of standard stock solution. The amount of the starting material was calculated with reference to the internal standard and K value. Then, the mass of both starting material and product was calculated before converting to moles for the conversion.

ICP-OES Measurement Details

Approximately 100 mg of the product was extracted after reaction in flow system, introduced into PTFE vessels and topped up with 3 mL of Supra Pure nitric acid (65%). The vessels were capped and placed in a microwave digester to decompose the organic substances. The microwave was heated up to 180 °C at 500 W for 15-30 min. The temperature was then fixed at 180 °C and 500 W for 10 min. The digested substances were then left to cool to room temperature before transferring to a 25 mL or 50 mL volumetric flasks and topping up with ultra-pure water.

Standard calibration curve was plotted with commercially available standards which are diluted to 0, 1, 10, 100, 250, 500 and 1000 ppb. Ruthenium concentrations of the samples were then determined by ICP-OES from this standard calibration curve. The concentration obtained, in parts per billion (ppb), from ICP-OES was multiplied by the dilution factor of 25 and divided by the mass (measured beforehand). Example results: 9.094 ppb signal in the ICP-OES concluded that in 101.66 mg sample, there was 2.2 parts per million (ppm) of ruthenium present.

^{1}H and ^{13}C NMR Spectra

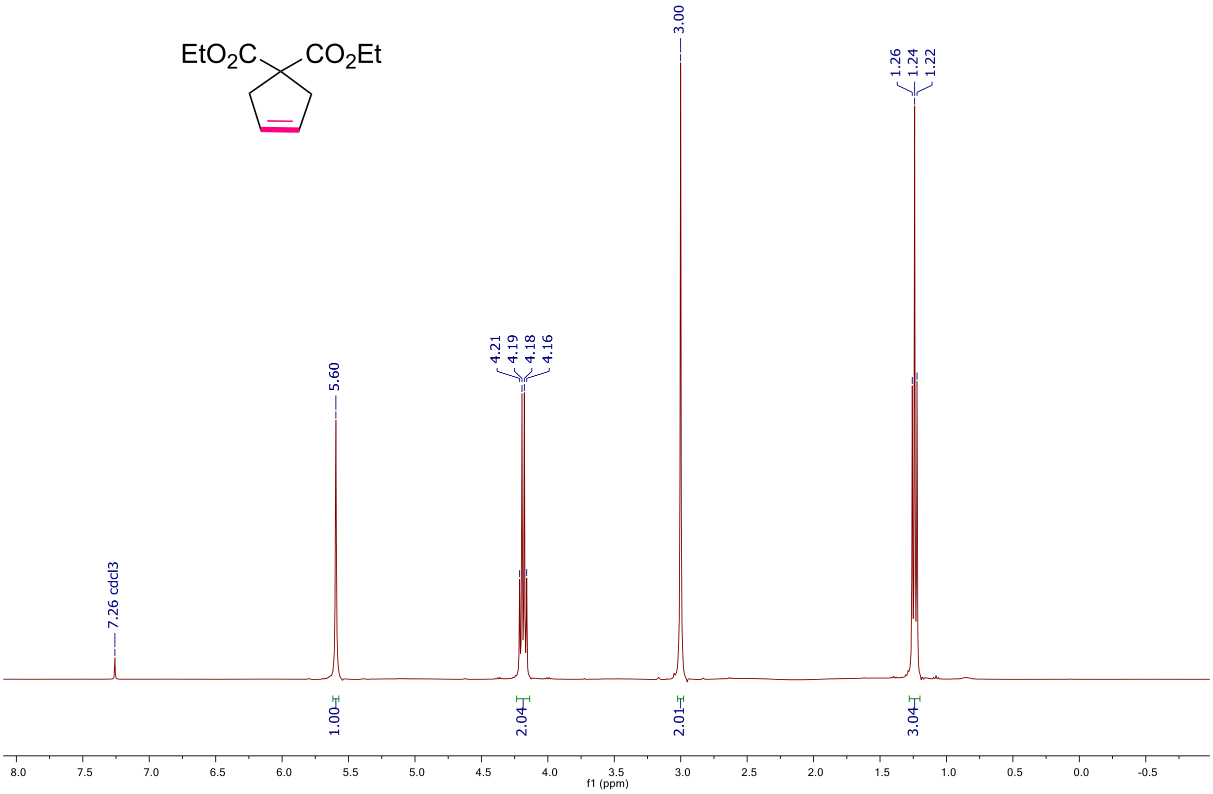

Figure S6. ^{1}H NMR of diethyl cyclopent-3-ene-1,1-dicarboxylate (**2**).

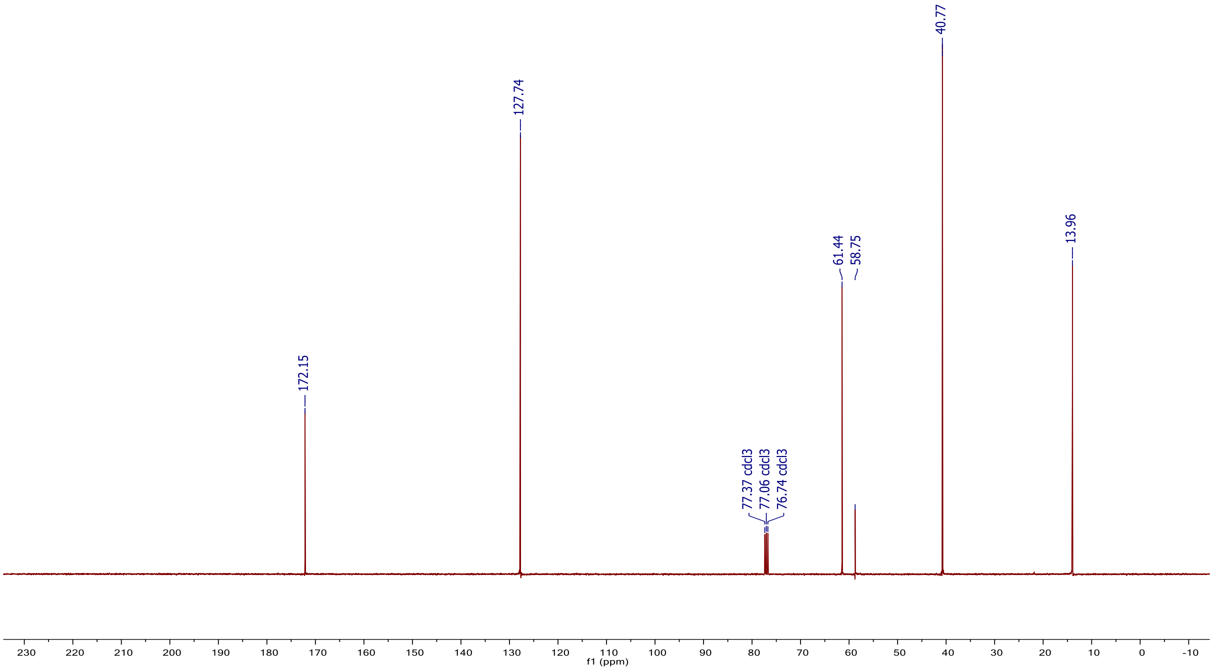

Figure S7. ^{13}C NMR of diethyl cyclopent-3-ene-1,1-dicarboxylate (**2**).

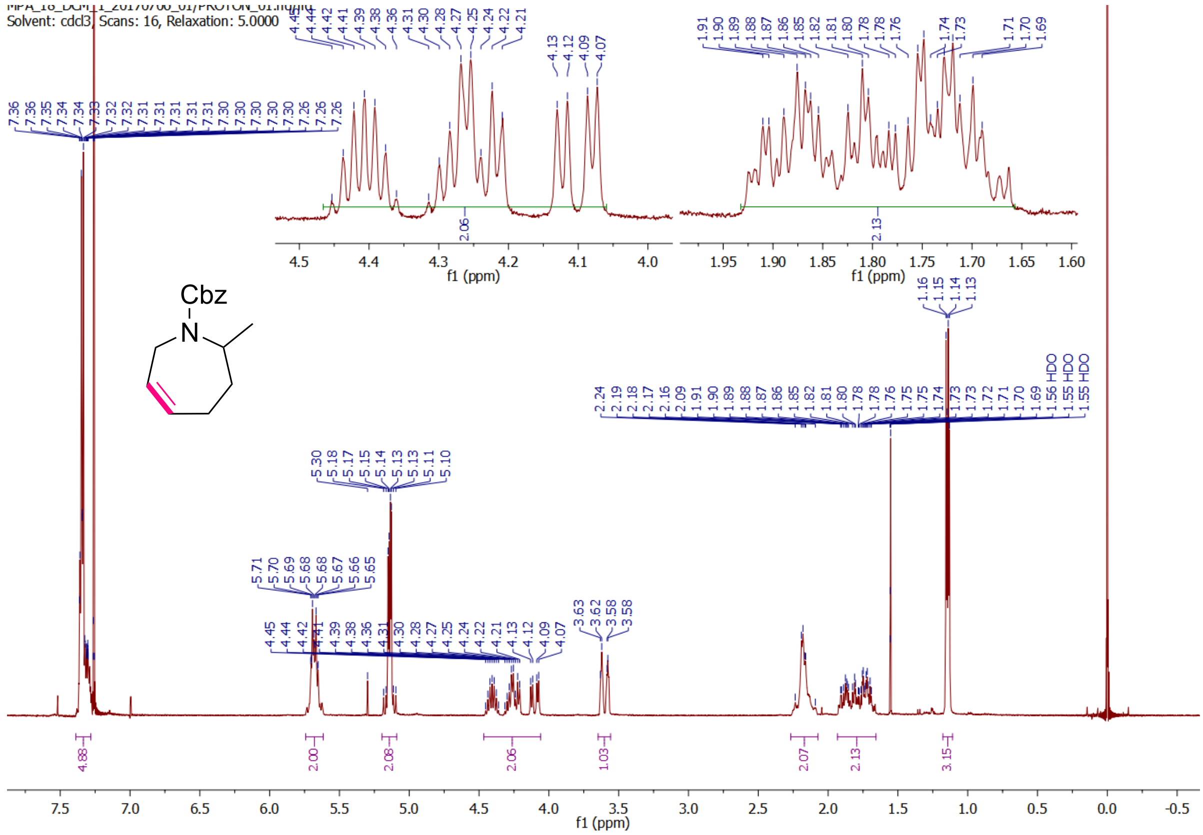

Figure S8. ^{1}H NMR of benzyl 2-methyl-2,3,4,7-tetrahydro-1*H*-azepine-1-carboxylate (**4**).

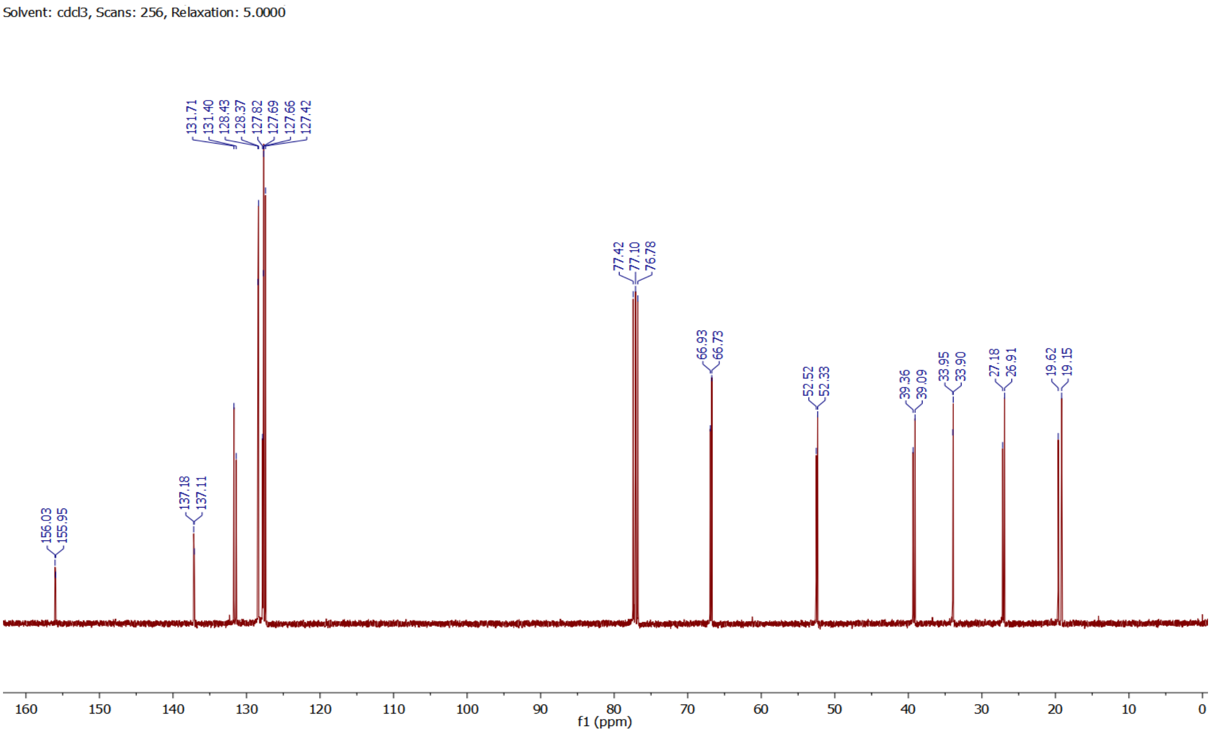

Figure S9. ^{13}C NMR of benzyl 2-methyl-2,3,4,7-tetrahydro-1*H*-azepine-1-carboxylate (**4**).

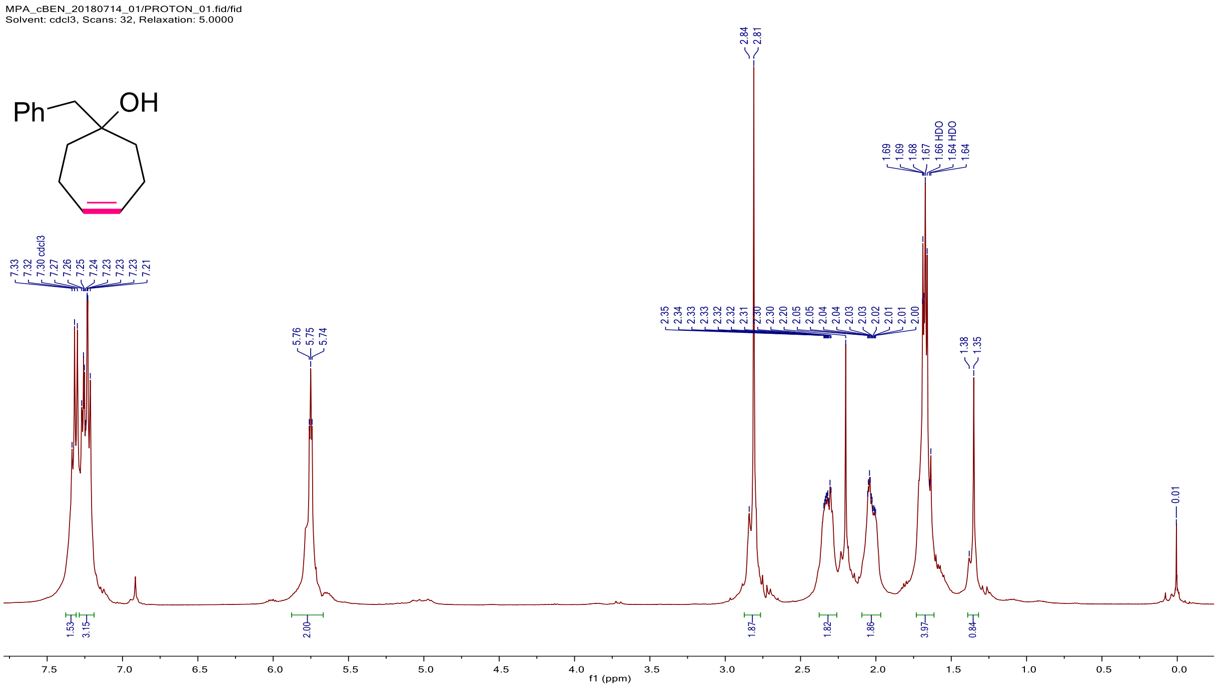

Figure S10. ^{1}H NMR of 1-benzylcyklohept-4-en-1-ol (**6**).

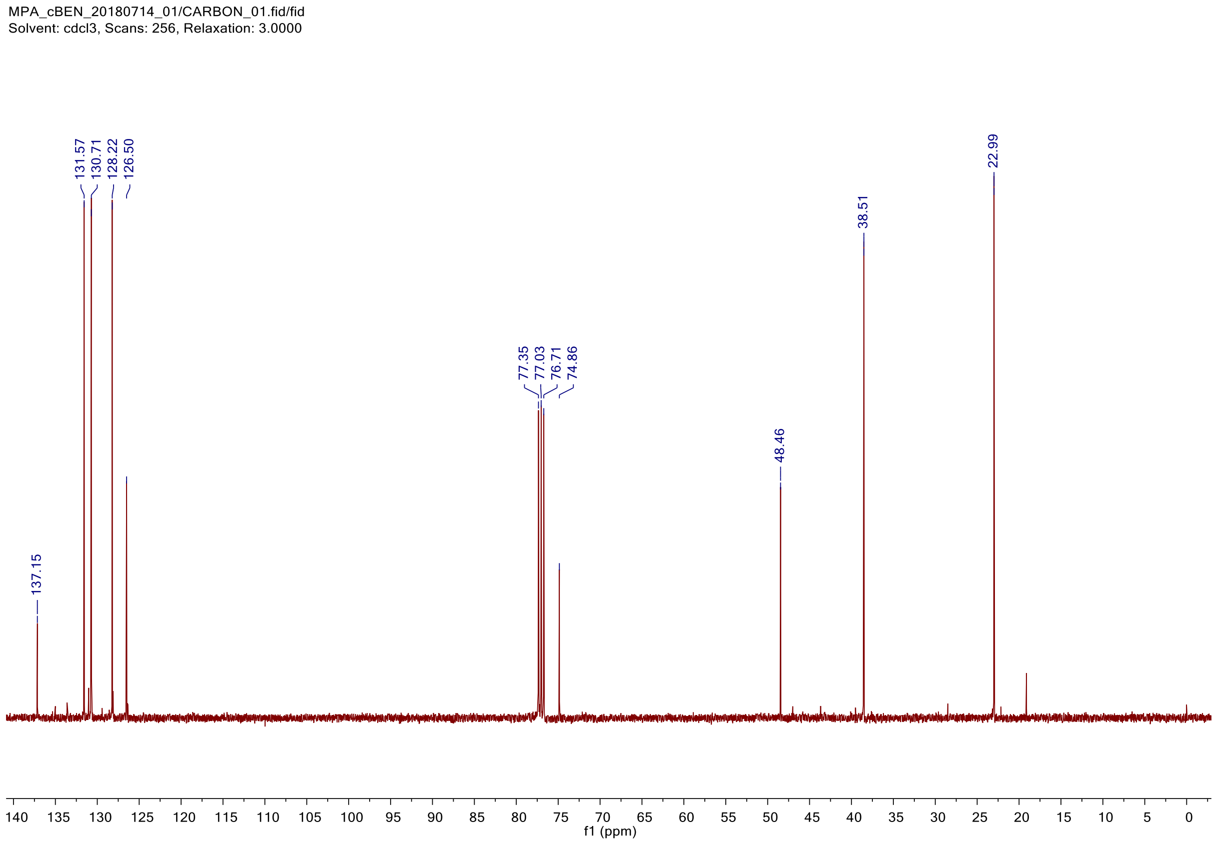

Figure S11. ^{13}C NMR of 1-benzylcyklohept-4-en-1-ol (**6**).

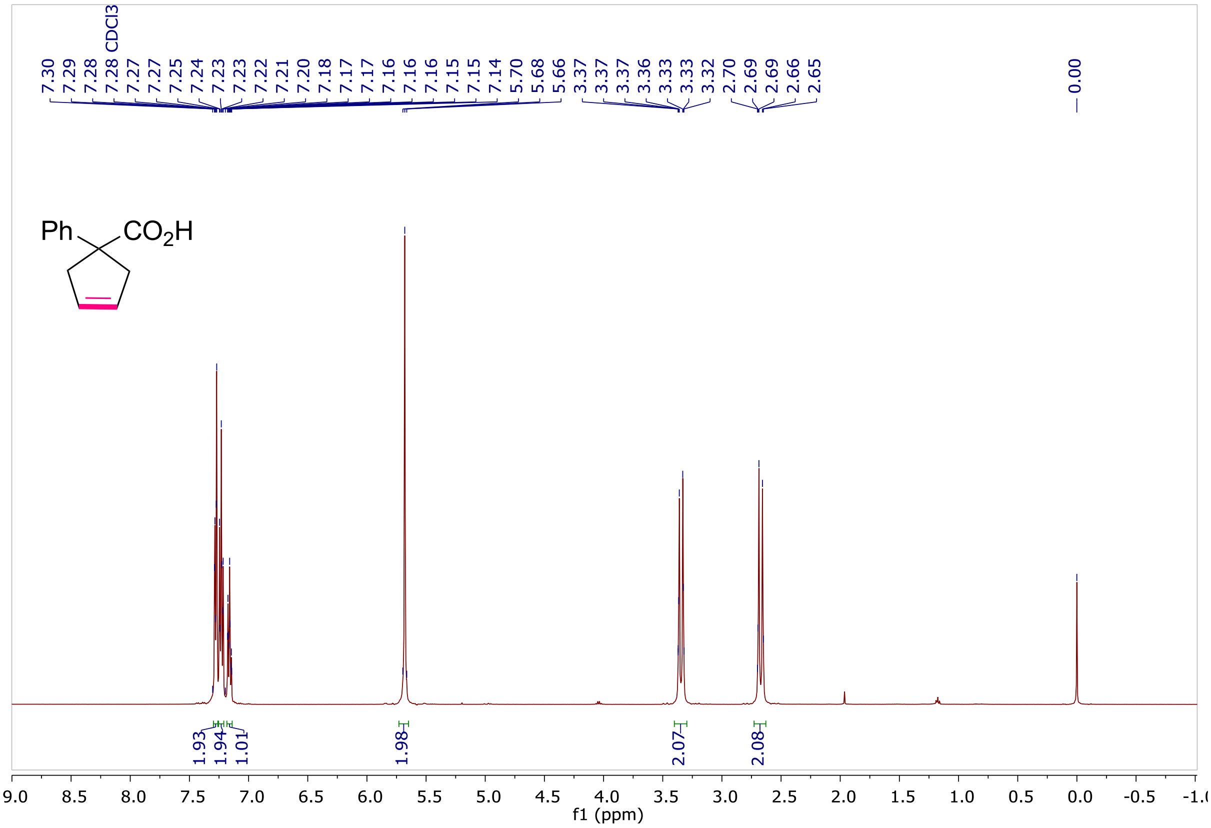

Figure S12. ^{1}H NMR of 1-phenylcyclopent-3-ene-1-carboxylic acid (**8**).

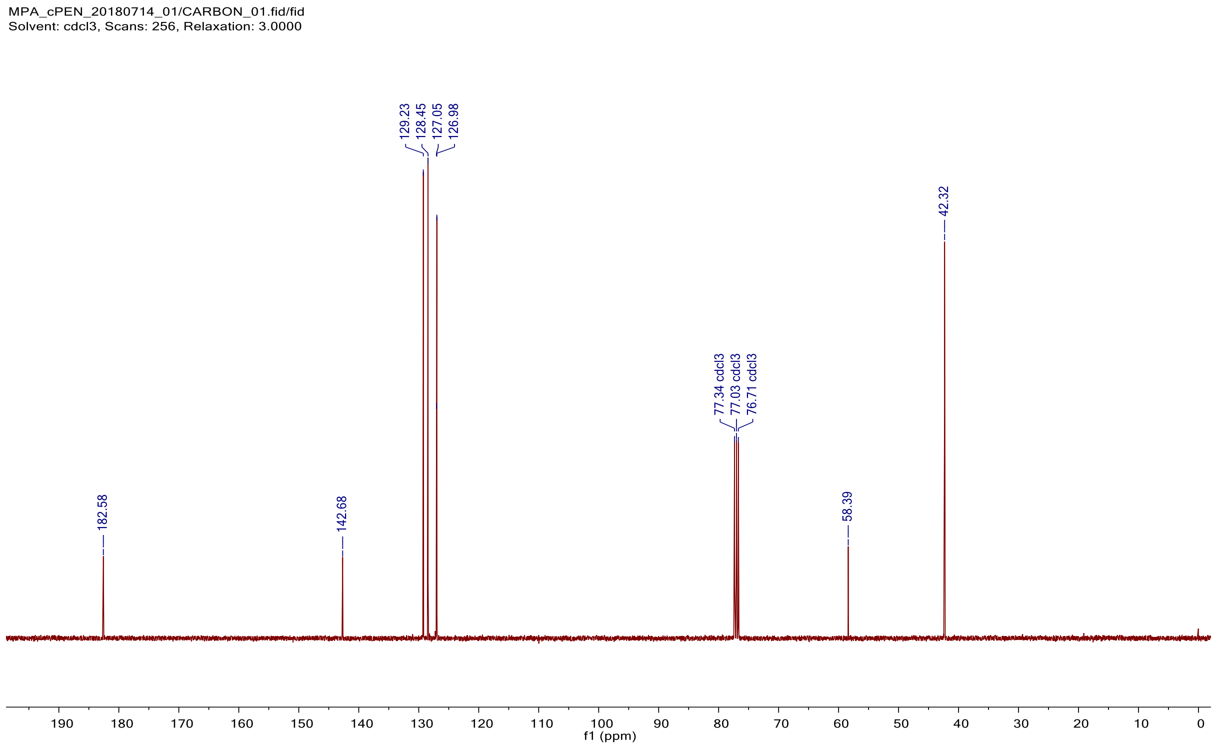

Figure S13. ^{13}C NMR of 1-phenylcyclopent-3-ene-1-carboxylic acid (**8**).

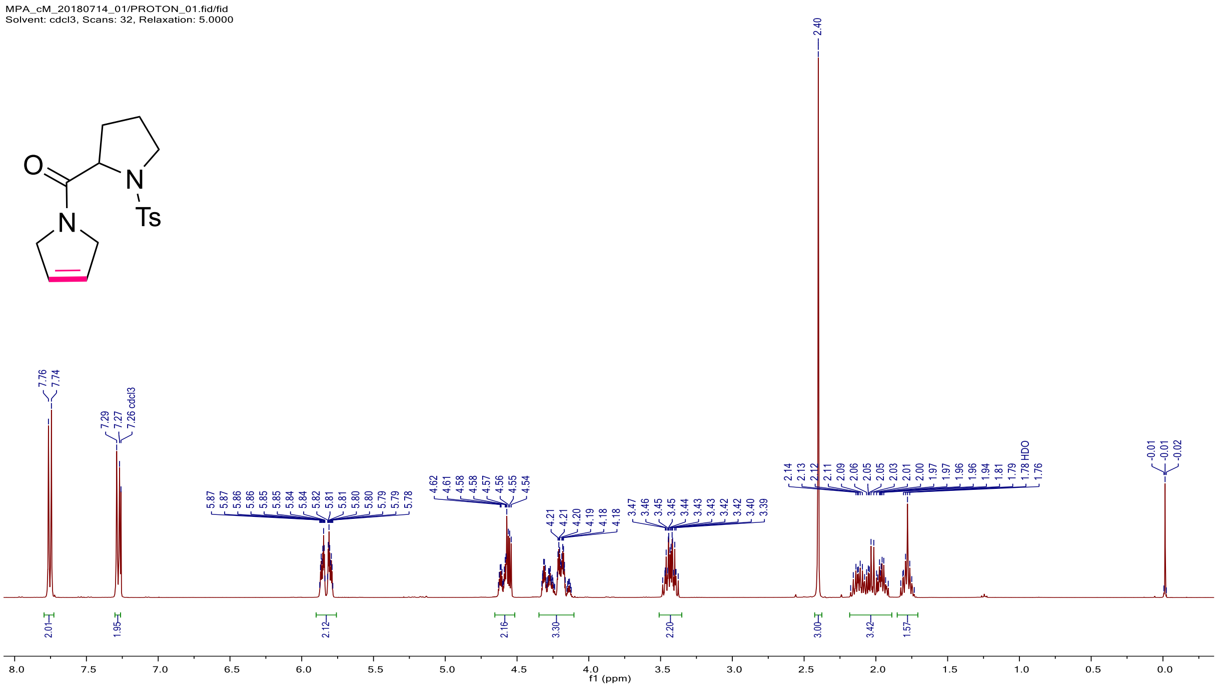

Figure S14. ^{1}H NMR of 1-(tosylpropyl)-2,5-dihydro-1*H*-pyrrole (**10**).

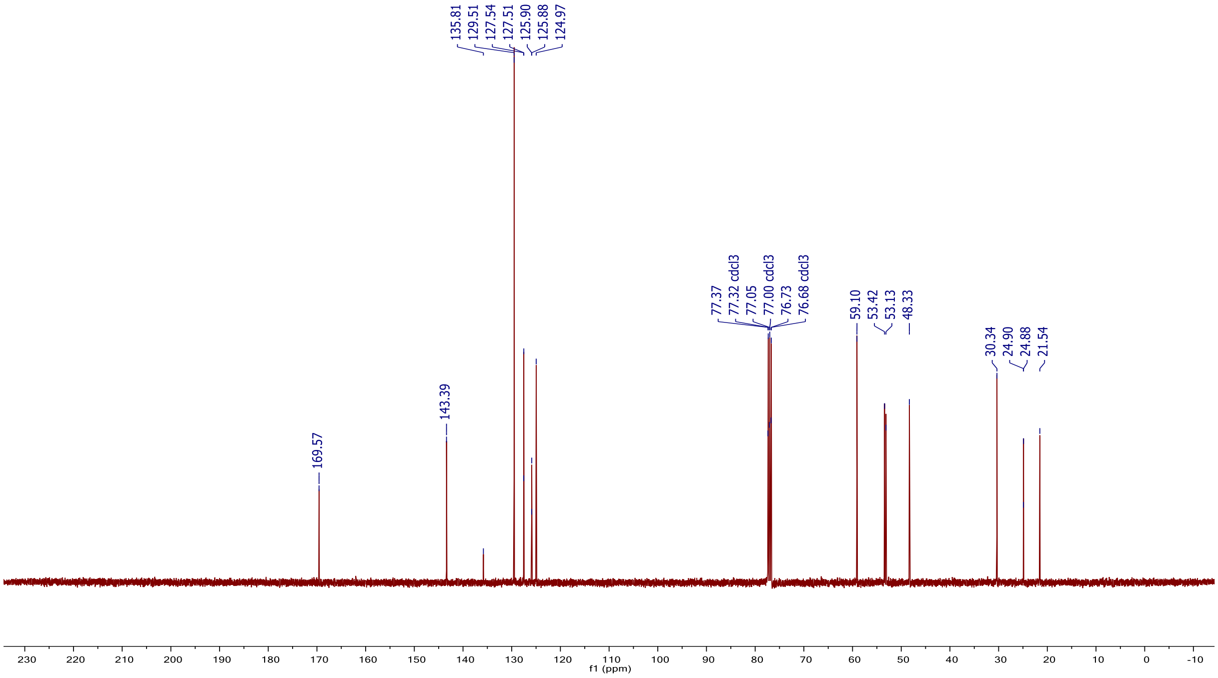

Figure S15. ^{13}C NMR of 1-(tosylpropyl)-2,5-dihydro-1*H*-pyrrole (**10**).

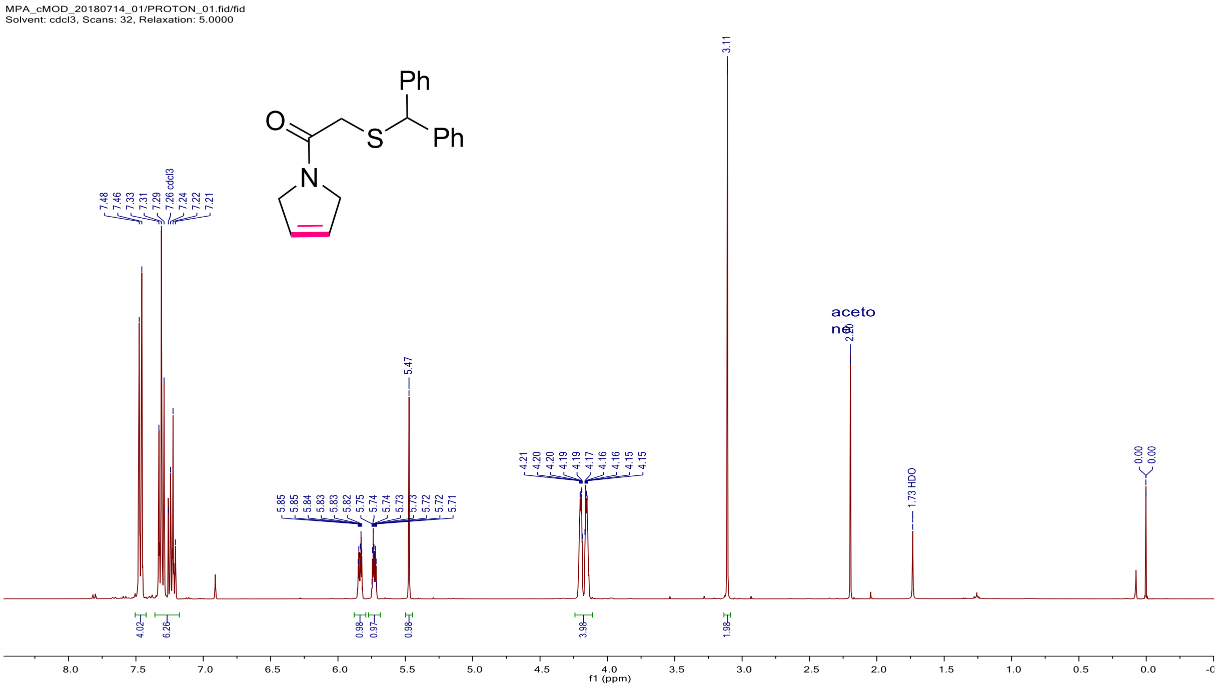

Figure S16. ^{1}H NMR of 2-(benzhydrylthio)-1-(2,5-dihydro-1*H*-pyrrol-1-yl)ethan-1-one (**12**).

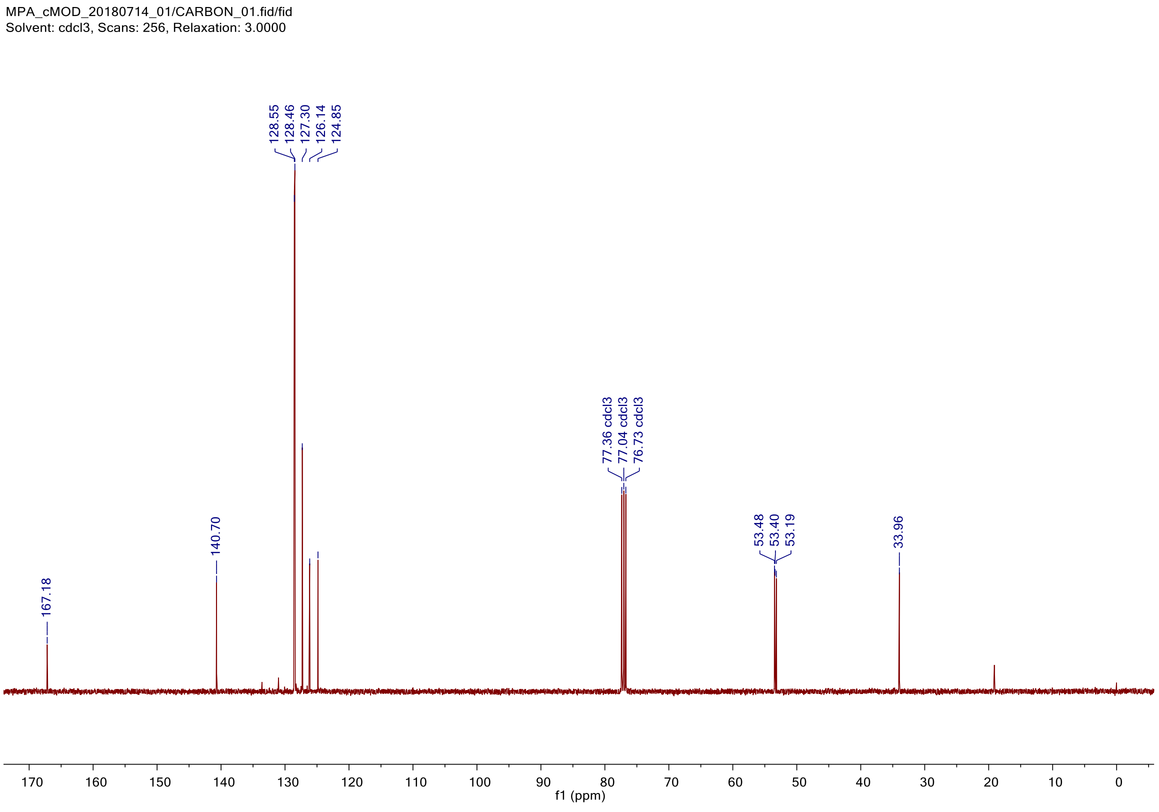

Figure S17. ^{13}C NMR of 2-(benzhydrylthio)-1-(2,5-dihydro-1*H*-pyrrol-1-yl)ethan-1-one (**12**).

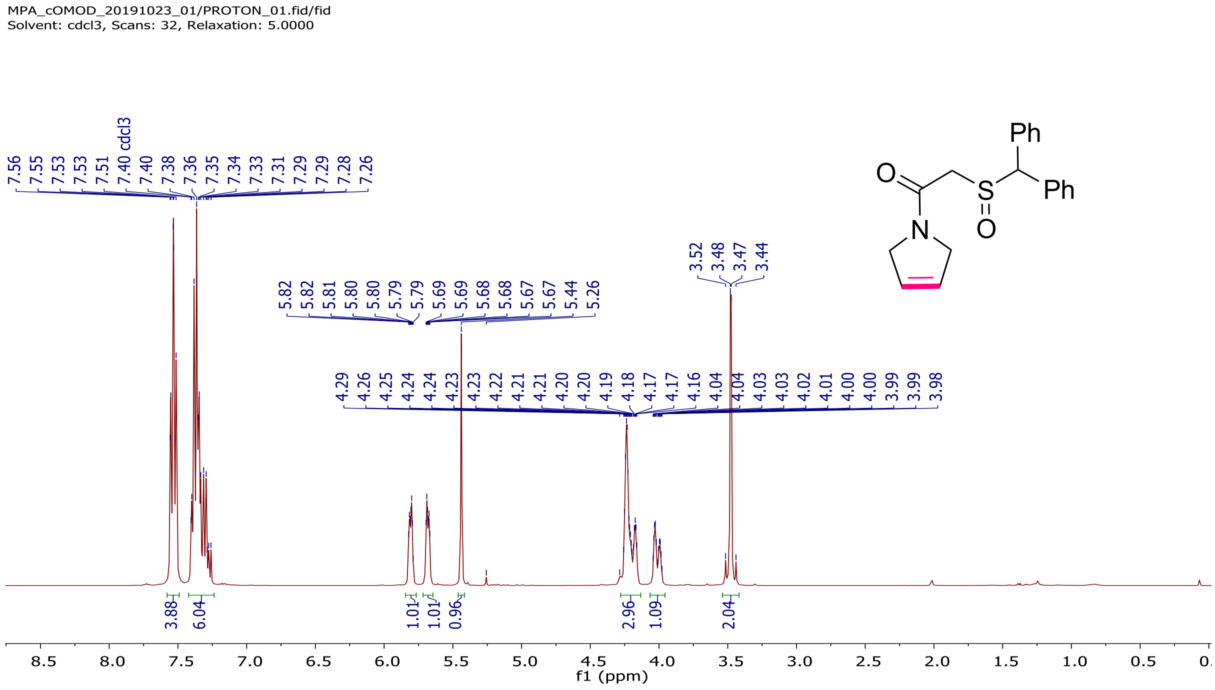

Figure S18. ^{1}H NMR of 2-(benzhydrylsulfinyl)-1-(2,5-dihydro-1*H*-pyrrol-1-yl)ethan-1-one (**14**).

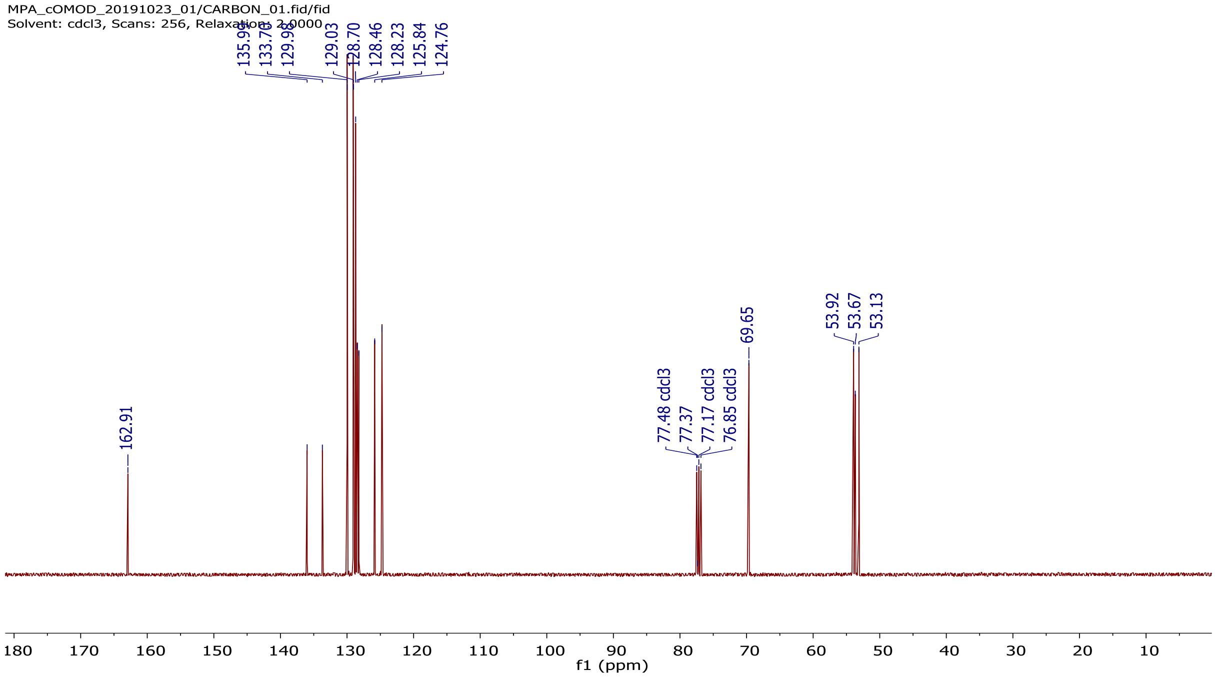

Figure S19. ^{13}C NMR of 2-(benzhydrylsulfinyl)-1-(2,5-dihydro-1*H*-pyrrol-1-yl)ethan-1-one (**14**).

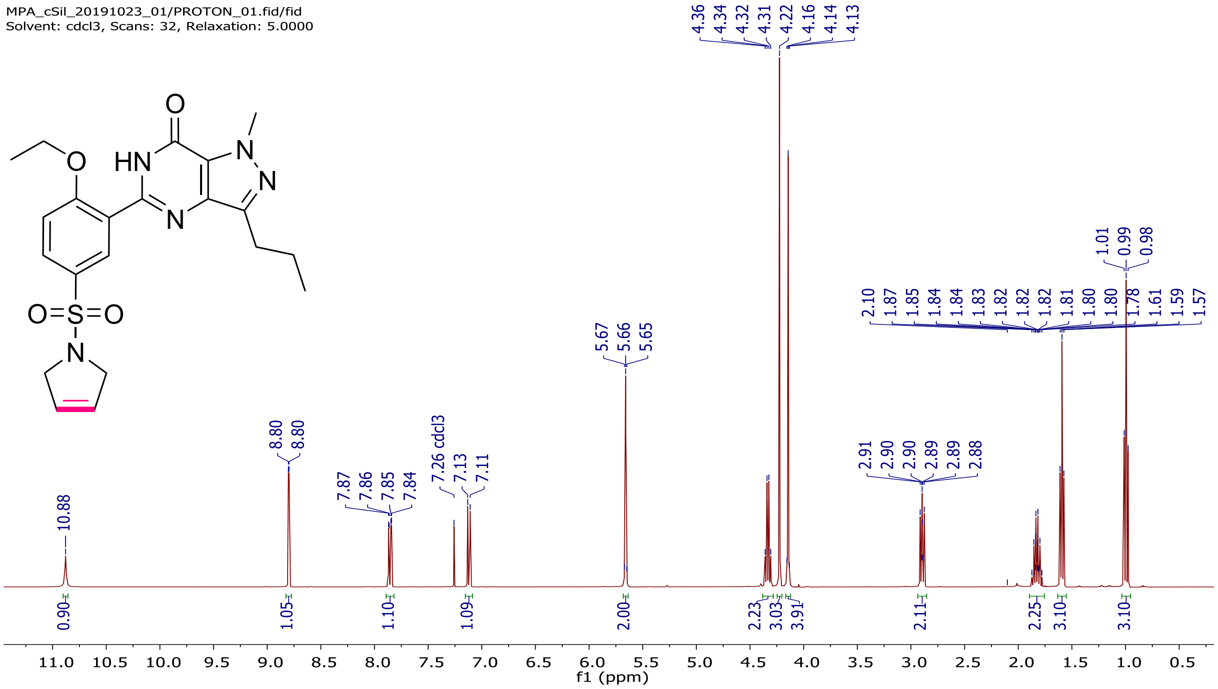

Figure S20. ^{1}H NMR of 5-(5-((2,5-dihydro-1*H*-pyrrol-1-yl)sulfonyl)-2-ethoxyphenyl)-1-methyl-3-propyl-1,6-dihydro-7*H*-pyrazolo[4,3-d]pyrimidin-7-one (**16**).

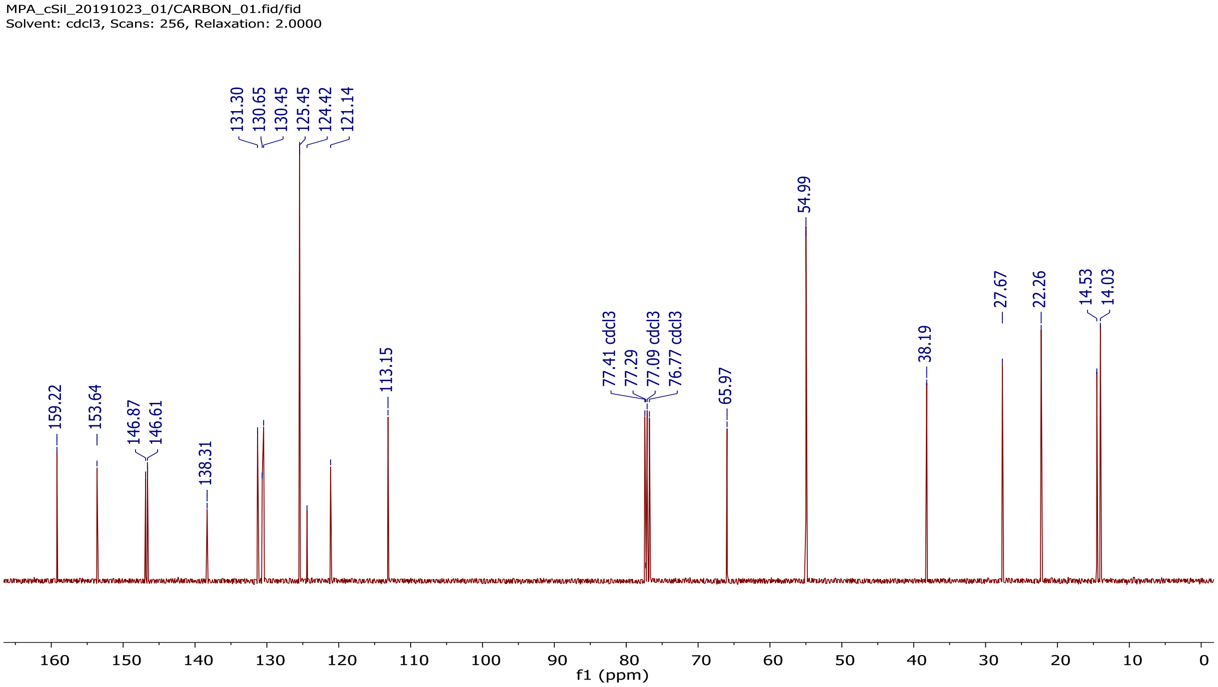

Figure S21. ^{13}C NMR of 5-(5-((2,5-dihydro-1*H*-pyrrol-1-yl)sulfonyl)-2-ethoxyphenyl)-1-methyl-3-propyl-1,6-dihydro-7*H*-pyrazolo[4,3-d]pyrimidin-7-one (**16**).

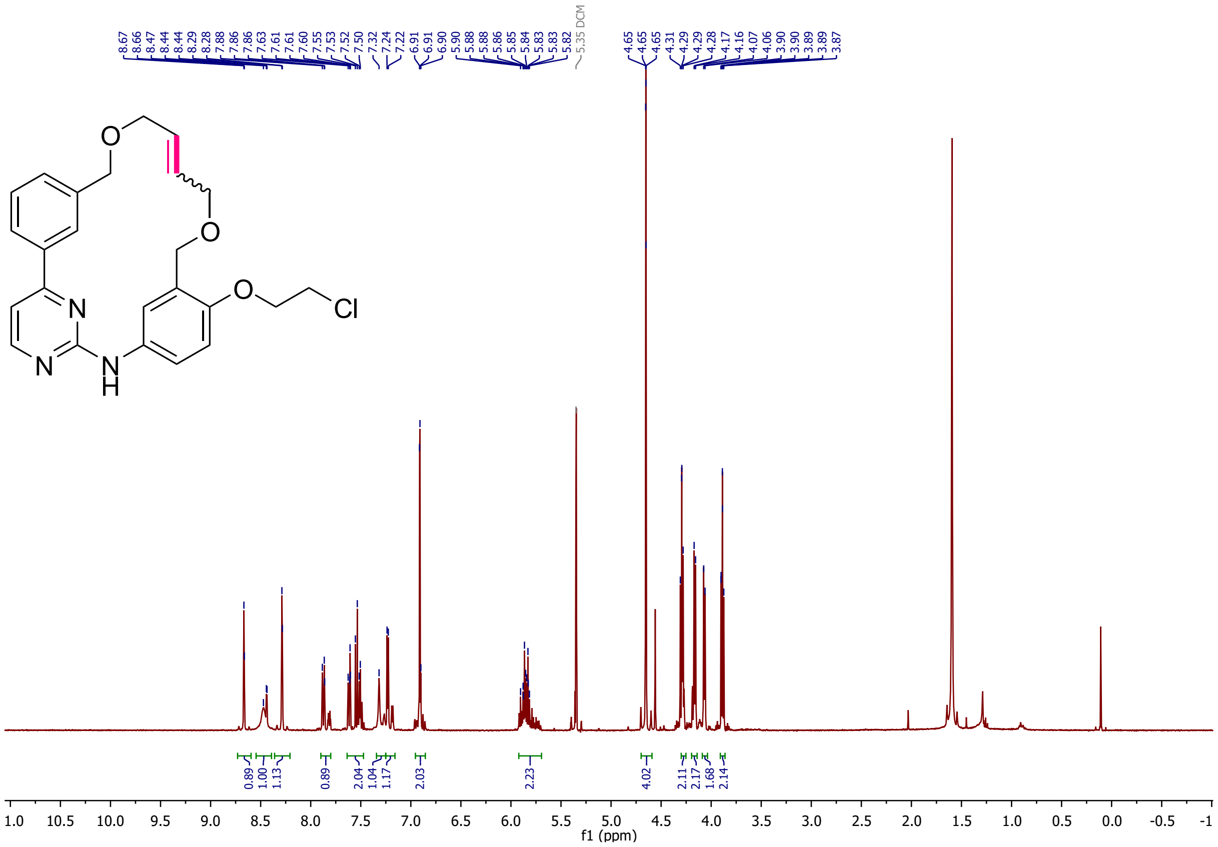

Figure S22. ^{1}H NMR of 4[4]-(2-chloroethoxy)-6,11-dioxa-3-aza-2(4,2)-pyrimidina-1,4(1,3)-dibenzenacyclododecaphan-8-ene (**18**).

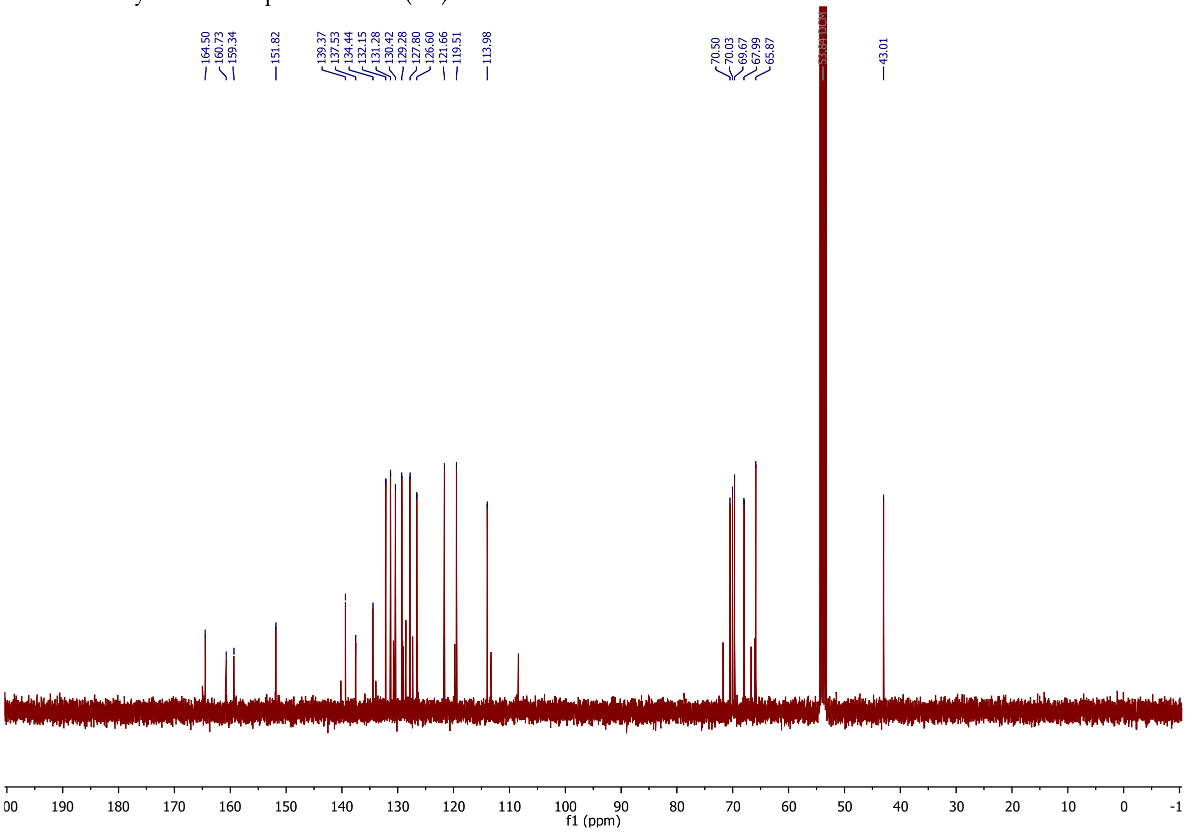

Figure S23. ^{13}C NMR of 4[4]-(2-chloroethoxy)-6,11-dioxa-3-aza-2(4,2)-pyrimidina-1,4(1,3)-dibenzenacyclododecaphan-8-ene (**18**).

References

1. Taber, D. F.; Frankowski, K. J., Grubbs' Catalyst in Paraffin: An Air-Stable Preparation for Alkene Metathesis. *The Journal of Organic Chemistry* **2003,** *68* (15), 6047-6048. DOI: 10.1021/jo030005p.

2. Szczepaniak, G.; Nogaś, W.; Piątkowski, J.; Ruszczyńska, A.; Bulska, E.; Grela, K., Semiheterogeneous Purification Protocol for the Removal of Ruthenium Impurities from Olefin Metathesis Reaction Products Using an Isocyanide Scavenger. *Organic Process Research & Development* **2019,** *23* (5), 836-844. DOI: 10.1021/acs.oprd.8b00392.

3. Rodriguez, S.; Uria, U.; Reyes, E.; Carrillo, L.; Tejero, T.; Merino, P.; Vicario, J. L., Enantioselective Synthesis of Tropanes: Brønsted Acid Catalyzed Pseudotransannular Desymmetrization. *Angewandte Chemie International Edition* **2020,** *59* (17), 6780-6784. DOI: 10.1002/anie.202000650.

4. Chołuj, A.; Krzesiński, P.; Ruszczyńska, A.; Bulska, E.; Kajetanowicz, A.; Grela, K., Noncovalent Immobilization of Cationic Ruthenium Complex in a Metal–Organic Framework by Ion Exchange Leading to a Heterogeneous Olefin Metathesis Catalyst for Use in Green Solvents. *Organometallics* **2019,** *38* (18), 3397-3405. DOI: 10.1021/acs.organomet.9b00287.

5. Małecki, P.; Gajda, K.; Gajda, R.; Woźniak, K.; Trzaskowski, B.; Kajetanowicz, A.; Grela, K., Specialized Ruthenium Olefin Metathesis Catalysts Bearing Bulky Unsymmetrical NHC Ligands: Computations, Synthesis, and Application. *ACS Catalysis* **2019,** *9* (1), 587-598. DOI: 10.1021/acscatal.8b04783.

6. Żukowska, K.; Pączek, Ł.; Grela, K., Sulfoxide-Chelated Ruthenium Benzylidene Catalyst: a Synthetic Study on the Utility of Olefin Metathesis. *ChemCatChem* **2016,** *8* (17), 2817-2823. DOI: 10.1002/cctc.201600538.

7. Nienałtowski, T.; Krzesiński, P.; Baumert, M. E.; Skoczeń, A.; Suska-Kauf, E.; Pawłowska, J.; Kajetanowicz, A.; Grela, K., 4-Methyltetrahydropyran as a Convenient Alternative Solvent for Olefin Metathesis Reaction: Model Studies and Medicinal Chemistry Applications. *ACS Sustainable Chemistry & Engineering* **2020,** *8* (49), 18215-18223. DOI: 10.1021/acssuschemeng.0c06668.

8. William, A. D.; Lee, A. C. H.; Blanchard, S.; Poulsen, A.; Teo, E. L.; Nagaraj, H.; Tan, E.; Chen, D.; Williams, M.; Sun, E. T.; Goh, K. C.; Ong, W. C.; Goh, S. K.; Hart, S.; Jayaraman, R.; Pasha, M. K.; Ethirajulu, K.; Wood, J. M.; Dymock, B. W., Discovery of the Macrocycle 11-(2-Pyrrolidin-1-yl-ethoxy)-14,19-dioxa-5,7,26-triaza-tetracyclo[19.3.1.1(2,6).1(8,12)]heptacosa-1(25),2(26),3,5,8,10,12(27),16,21,23-decaene (SB1518), a Potent Janus Kinase 2/Fms-Like Tyrosine Kinase-3 (JAK2/FLT3) Inhibitor for the Treatment of Myelofibrosis and Lymphoma. *Journal of Medicinal Chemistry* **2011,** *54* (13), 4638-4658. DOI: 10.1021/jm200326p.
